# The impact of long-term care interventions on healthcare utilisation among older persons: a scoping review of reviews

**DOI:** 10.1186/s12877-024-05097-9

**Published:** 2024-06-03

**Authors:** Nur Zahirah Balqis-Ali, Suhana Jawahir, Yee Mang Chan, Amanda Wei-Yin Lim, Ummi Wahidah Azlan, Sal Sabila Mohd Shaffie, Weng Hong Fun, Shaun Wen Huey Lee

**Affiliations:** 1 Institute for Health Systems Research, National Institutes of Health, Ministry of Health, Shah Alam, Selangor Malaysia; 2 Institute for Public Health, National Institutes of Health, Ministry of Health, Shah Alam, Selangor Malaysia; 3 Institute for Clinical Research, National Institutes of Health, Ministry of Health, Shah Alam, Selangor Malaysia; 4 School of Pharmacy, Monash University Malaysia, Subang Jaya, Selangor Malaysia; 5 School of Pharmacy, Taylor’s University Lakeside Campus Malaysia, Subang Jaya, Selangor Malaysia

**Keywords:** Long-term care, Older persons, Healthcare utilisation, Scoping review

## Abstract

**Background:**

As the ageing population grows, the demand for long-term care (LTC) services will rise, concurrently amplifying healthcare utilisation. This review aims to examine and consolidate information on LTC interventions that influence healthcare utilisation among older persons.

**Methods:**

A scoping review was performed through a systematic search in PubMed, EBSCO CINAHL, EBM Reviews - Cochrane Database of Systematic Reviews, Embase, APA PsycInfo, EBM Reviews - Health Technology Assessment, and EBM Reviews - NHS Economic Evaluation Database. Systematic reviews with meta-analyses published between 1 January 2010 and 2 June 2022 among older persons aged 60 and above were included. The characteristics of LTC interventions were mapped to the World Health Organization (WHO) Healthy Ageing Framework. The effect sizes of healthcare utilisations for LTC interventions were recalculated using a random-effects model. The methodological quality was assessed with the AMSTAR-2 checklist, while the quality of evidence for each association was evaluated using GRADE.

**Results:**

Thirty-seven meta-analyses were included. The most prominent domain of the healthy ageing framework was managing chronic conditions. One hundred twelve associations between various LTC interventions and healthcare utilisations were identified, with 22 associations impacting healthcare utilisation. Four interventions were supported by suggestive or convincing evidence. Preventive home visits were found to reduce hospital admission (OR: 0.73, 95% CI: 0.59, 0.91, *p* = 0.005), caregiver integration during discharge planning (OR: 0.68, 95% CI: 0.57, 0.81, *p* < 0.001), and continuity of care (OR: 0.76, 95% CI: 0.61, 0.95, *p* = 0.018) reduced hospital readmission, and perioperative geriatric interventions reduced the length of hospital stay (MD: -1.50, 95% CI: -2.24, -0.76, *p* < 0.001). None of the associations impacted emergency department visits, medication use, and primary care utilisations with convincing evidence. Most reviews received low methodological quality.

**Conclusion:**

The findings suggest that LTC interventions could benefit from transitioning to a community-based setting involving a multidisciplinary team, including carers. The spectrum of services should incorporate a comprehensive assessment to ensure continuous care.

**Supplementary Information:**

The online version contains supplementary material available at 10.1186/s12877-024-05097-9.

## Background

Population ageing is a global phenomenon, with the number of older persons projected to double from 771 million in 2022 to 1.6 billion in 2050 [1]. Low—and middle-income countries (LMICs) are projected to experience the most significant change, with nearly 80% of the world’s population over 60 living in LMICs by 2050 [2]. This demographic shift is expected to transform societies across many spectrums, impacting healthcare systems, social welfare programs, economic productivity, and family structures [3].

As the older population continues to increase, there will be a corresponding rise in the demand for long-term care (LTC) services, encompassing home and community-based care, healthcare monitoring, rehabilitation, and therapy services. These services are defined as those that safeguard older persons’ intrinsic capacities and functional ability, ensuring they align with their fundamental rights, basic freedoms, and human dignity [4, 5]. As the healthcare system shifts away from being disease-based and evolves towards holistic and comprehensive care, the importance of LTC services in supporting older persons becomes increasingly acknowledged and emphasised [6]. In response, the World Health Organization (WHO) identified a need to promote health, prevent disease, maintain intrinsic capacity, and enable the functional ability of older persons by ensuring access to LTC [5]. The WHO has developed a public health framework for healthy ageing comprising three domains: health services, LTC, and environments [7]. These domains encompass various aspects of healthcare, such as preventing chronic conditions, facilitating early detection and control, reversing or mitigating declines in capacity, managing advanced chronic conditions, and promoting capacity-enhancing behaviours.

Evidence suggests that diminished functional ability in older persons correlates with increased utilisation of healthcare services, leading to higher treatment costs and a greater likelihood of institutionalisation [8–10]. Despite some progress in the formal delivery of LTC services in many LMICs, family members or caregivers continue to shoulder the bulk of LTC needs [6]. Thus, there exists a pressing need to integrate LTC into health systems delivery to ensure that services are readily accessible to support and prevent functional decline among older persons [11]. The WHO has developed guiding frameworks and models to facilitate the seamless integration of LTC into health system policies, promoting accessibility and efficacy in care delivery [12]. However, incorporating an effective LTC system is complex, often involving commitments across diverse care settings. In many LMICs, policymakers encounter the challenge of aligning LTC within broader health system perspectives, usually contending with limited resources and conflicting priorities [13, 14]. Therefore, it is crucial to identify, map, and summarise the global LTC interventions and services for older persons while considering their influence on healthcare utilisation.

While substantial evidence exists regarding the effectiveness of various LTC interventions [15–18], there are conflicting findings. To our knowledge, few studies have mapped the characteristics of LTC interventions [19, 20], but the impact of LTC interventions on healthcare utilisation is unknown. Owing to the abundance of knowledge on LTC interventions, we augmented and advanced the existing knowledge through a comprehensive scoping review focused on systematic reviews with meta-analyses. The primary objective is to examine and consolidate information on LTC interventions that influence healthcare utilisation among older persons. These findings are pivotal in guiding policy development, particularly in identifying and prioritising LTC services that positively contribute to the healthcare system and improve the overall care for older persons.

## Methods

A scoping review was reported based on the methodological framework for scoping studies by Arksey and O’Malley [21] and Preferred Reporting Items for Systematic Review and Meta-analyses (PRISMA) guidelines [22]. The research protocol was registered as part of a more extensive study (Trial registration: NMRR-21-467-58076) and in the Open Science Framework (OSF) [23]. Due to the extent of the study scope and search, the study was amended from an umbrella review to a scoping review. While an umbrella review typically addresses a narrower research question, often focusing on specific interventions or outcomes [24], the current study encompasses a broader range of both interventions and outcomes. Therefore, it was determined that a scoping review would be a more appropriate methodology based on the research focus.

The scoping review specifically targeted systematic reviews accompanied by meta-analyses, delineated as articles explicitly identified as such in their title, abstract, or methods section. This allows the examination of a range of heterogeneous interventions that could be aggregated to assess and quantify their collective impact on healthcare utilisation. To provide a comprehensive overview of interventions considered in the meta-analysis, individual trials not pooled into meta-analyses in the articles were retained in this study.

### Stage 1: identifying the research question

The scoping review aimed to address the following question: What insights does the existing systematic review with meta-analyses offer regarding the impact of LTC interventions on healthcare utilisation among older persons?

### Stage 2: identifying relevant studies

A systematic search was performed on the following databases: PubMed, EBSCO CINAHL Plus, Cochrane Database Systematic Review, Embase, APA PsychINFO, EBM Reviews - Health Technology Assessment, and EBM Reviews - NHS Economic Evaluation Database. The search included Medical Subject Headings (MeSH) terms supplemented with a search of reference lists from identified studies (Additional file 1). The initial search occurred in November 2021, with three updated searches in June 2022, May 2023, and April 2024.

### Stage 3: study selection

#### Inclusion criteria

Studies were eligible for inclusion if they were: a) Systematic reviews with meta-analyses encompassing Randomised Controlled Trials (RCTs) and observational studies investigating both single and multi-component LTC interventions or services; b) included older persons aged 60 years and above; c) targeted interventions or services in any setting, including home, community, healthcare facility, nursing homes or residential aged care facility; d) reported on health service utilisation as an outcome; and e) published between January 2010 and June 2022 in English. This study defined LTC as a wide range of interventions and services, such as managing chronic geriatric conditions, rehabilitation, palliation, promotion, and preventative services [25]. The search was limited to the year 2010 onwards to allow for the identification of recent evidence. As the aim of this study was to support health systems planning, only healthcare service utilisation reported from a health systems perspective was included, such as a) Hospital utilisation, b) Emergency department (ED) utilisation, c) Medication utilisation, and d) Primary care utilisation [26].

#### Exclusion criteria

Studies were excluded if: a) they included a disease-specific population; b) the target participants were not exclusively older persons and included a mixture of younger (below 60 years old) and older persons; c) reported outcomes focused exclusively on caregivers and/or health providers; d) reported on patient outcomes such as health-related quality of life; and e) focused exclusively on outcome measures for economic evaluation.

#### Screening and selection process

The selected studies were exported to a reference manager (EndNote X9) and deduplicated. Two reviewers independently screened the citation titles and abstracts for inclusion. The full text of the identified articles was retrieved and screened against the inclusion/exclusion criteria by another two independent reviewers. Any disagreements were resolved by consensus with a third reviewer.

### Stage 4: charting the data

Two pairs of reviewers then independently extracted the included studies using a standardised, pre-piloted data extraction form. The extracted information included study demographics, information related to primary studies included in the review, and LTC interventions/services. Summary findings were recorded in Excel Microsoft Office 365 (Additional file 2).

### Data synthesis and analysis

The results of the study were first described narratively. The LTC interventions were given a code based on the objective of the intervention in preventing or managing older persons’ intrinsic capacities or functional abilities. The coded LTC interventions were then mapped into several domains according to the WHO Healthy Ageing Framework [7], namely prevention activities, detection and control activities, management of chronic diseases, promotion and support of capacity-enhancing behaviours, ensuring a dignified late life, removing barriers to participation, and compensation for the loss of capacity by three independent reviewers. If needed, two other reviewers discussed any discrepancies and disagreements regarding the adjudication.

Interventions were then grouped into the four primary outcomes: hospital, emergency department, medication, and primary care utilisations and subdivided into separate domains. Hospital utilisation was further split into hospital admission, hospital readmission, length of stay or bed days. Emergency department (ED) utilisation was divided into ED visit, ED revisit, and length of stay. Medication utilisation refers to the number of drug use, and primary care utilisation refers to the number of visits.

Values extracted from all articles were reanalysed to standardise the findings, considering that various articles reported results in different units of measurement. Values were extracted across all interventions mentioned in the articles, irrespective of their inclusion in either meta-analyses or standalone analyses within the article. Trials from separate meta-analyses with similar intervention characteristics were analysed together unless the setting or follow-up duration differed. Redundant trials across different meta-analyses were removed, except in several situations whereby different values were extracted differently from the same trials. This discrepancy could arise from varying definitions of the outcomes among the authors or possibly from some authors reaching out to the primary author for supplementary data. The intervention durations were reclassified into four categories: less than six months, 7–12 months, 13–24 months, and 25–36 months for all outcomes. Consequently, the pooled interventions reported may deviate from the classification utilised in the original article. An illustration of the process flow is depicted in Additional File 3.

Due to the heterogeneity of included articles, RCTs and observational studies were analysed separately. Each association of long-term intervention with healthcare utilisations was reported in mean difference (MD) or odds ratio (OR) with a corresponding 95% confidence interval (CI) using the random-effects model, given the heterogeneity in design between and within studies [27]. The analysis was repeated using a fixed-effect model as a sensitivity analysis to investigate whether the method contributed to the observed high heterogeneity. When data from the articles were insufficient for reanalysis, we tried to contact the authors to gain the data. However, in cases where authors were not contactable, the data was extracted from the result as reported or marked as not reported (NR) when the data was unavailable. All statistical analyses were conducted with Stata version 14.0 (Stata Corp, College Station, TX, USA).

### Assessment of methodological quality

Two independent reviewers assessed the methodological quality of the included studies using the A Measurement Tool to Assess Systematic Reviews-2 (AMSTAR-2) checklist [28]. The index rates the quality of the studies based on seven critical and nine non-critical domains. Studies were rated high, moderate, low, and critically low quality. To aid in interpreting results, we assessed the quality of evidence of each outcome using the Grading of Recommendations, Assessment, Development, and Evaluations (GRADE) [29]. The quality of evidence was evaluated based on five domains, including a) risk of bias in individual studies b), inconsistency c), indirectness d), imprecision, and e) publication bias, subsequently classified as high, moderate, low, or very low quality. The quality ratings assigned to the evidence indicate the level of assurance in the accuracy of the estimated effects [30].

### Stage 5: collating, summarising, and reporting the results

All results were collated and summarised. The LTC interventions and their impacts on healthcare utilisation were presented.

### Ethics considerations

This scoping review was part of a more extensive study, ‘Simulation of Long-Term Care for Elderly in Malaysia’ (MyLTC, Trial registration number: NMRR-21-467-58076). The MyLTC protocol was approved by the Medical Research and Ethics Committee (MREC), Ministry of Health Malaysia. The study was conducted by Good Clinical Practice guidelines and the Declaration of Helsinki.

## Results

**Fig. 1:**
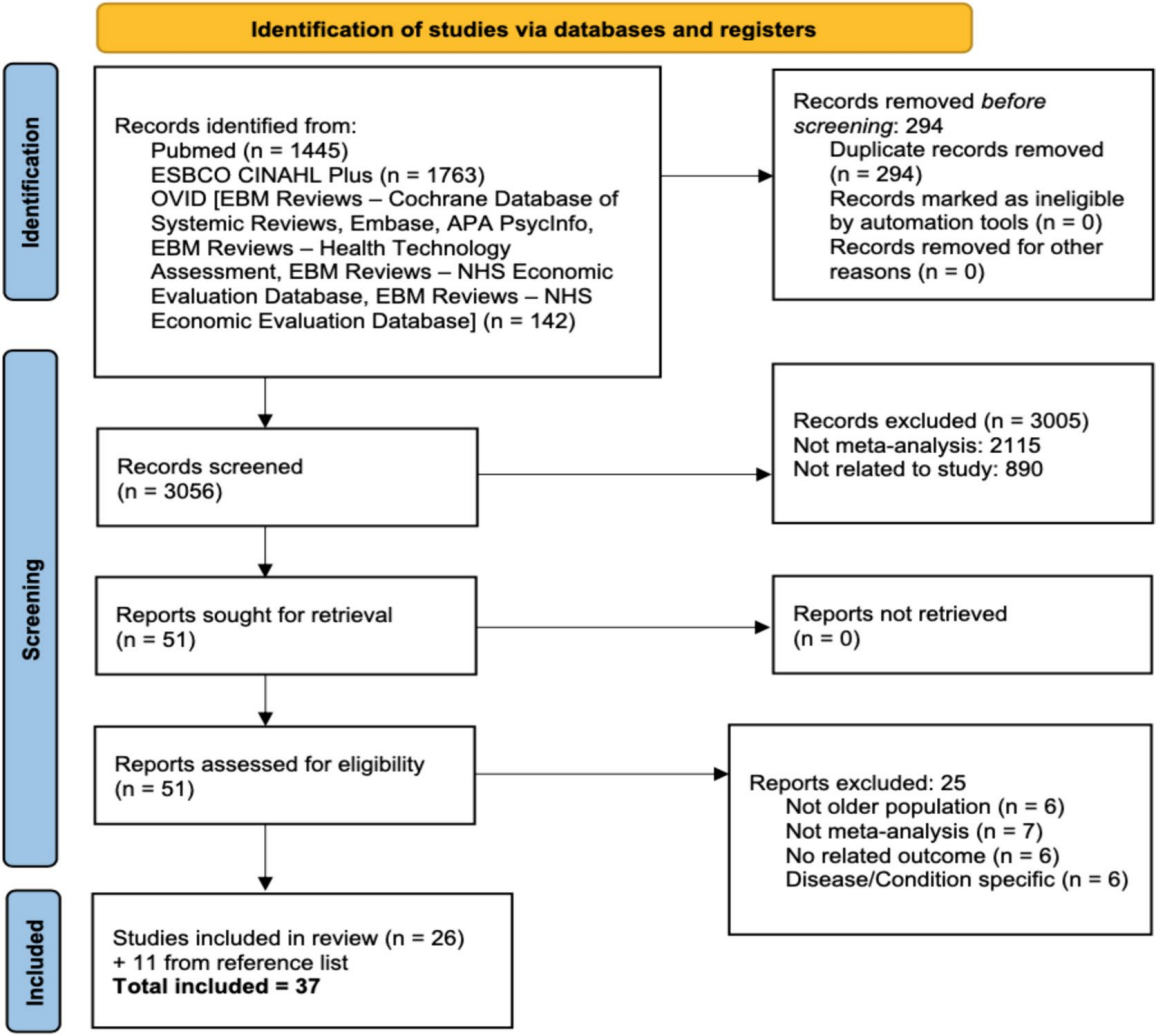
PRISMA 2020 flow diagram

The initial search identified 3,350 records, with 3,056 records screened after deduplication. Fifty-one full-text articles were screened, and 26 articles were selected after exclusion. An additional 11 studies were sourced from the reference list search. A total of 37 articles were included in this review (Fig. 1). The reasons for exclusion are provided in Additional File 4.

### Characteristics and methodological quality of articles exploring associations of long-term care interventions with healthcare utilisations for older persons

Of the 37 articles included, 17 were RCTs or cluster RCTs, while the remaining were mixed study designs. The median and interquartile range (IQR) for the number of studies per article was 17 [12–24]. The sample sizes ranged from 811 to the largest, involving 108,838 participants, with a median (IQR) of 9,679 (3,976 − 18,992). The duration of follow-up varies between studies, with the shortest follow-up within one week and the longest over 60 months. A total of 82 outcomes across all articles were identified. The most studied outcomes reported were hospital readmission (*n* = 19, 23.2%) and hospital admission (*n* = 18, 21.9%).

Most articles were rated either low or critically low in the methodological assessment using AMSTAR-2 (Table 1 and Additional file 5). This was mainly due to methodological issues, including the need for more justification for excluding individual studies and a lack of assessment regarding publication bias and its potential impact. The descriptive characteristics of the 37 eligible articles are provided in Table 1.

**Table 1:** Characteristics and methodological quality of articles included in the scoping review

Author	Design of included studies	Sample size (number of trials)	Population	Setting	Intervention	Outcomes Used in Scoping Review	AMSTAR-2 rating
Almutairi et al. (2020) [31]	RCT, cRCT	19,576 (25)	60 years or older; frail older persons	LTCF	Medication optimisation in residential aged/continuing care	● Hospital admission	Low
Beswick et al. (2010) [32]	RCT	108,838 (19)	65 years or older; living at home or preparing for hospital discharge to home.	Community	Community-based multifactorial interventions	● Hospital admissions	Critical low
Briggs et al. (2022) [33]	RCT, cRCT	7893 (21)	65 years or older; community-dwelling at risk of poor health outcomes	Community	CGA in a community setting	● Hospital admission ● ED visit	Critical low
Cochrane et al. (2016) [34]	RCT, cRCT, quasi-RCT	811 (2)	65 years or older; living in own home with poor physical and mental health	Community	Time-limited home-care reablement services	● Hospital admission ● ED visit	Moderate
Conroy et al. (2011) [35]	RCT	2287 (5)	65 years or older; frail older patients discharged rapidly (< 72 h) from an acute hospital setting	Acute care setting (ED)	CGA-post-hospital discharge model	● Hospital readmission	Critical low
Deschodt et al. (2013) [36]	nRCT, RCT, multicenter RCT	4546 (12)	60 years or older; hospitalised for at least 48 h on a nongeriatric ward	Hospital	CGA-consult	● Hospital readmission ● LOS	Critical low
Deschodt et al. (2020) [37]	RCT, prospective quasi-experimental	22,168 (19)	65 years or older; living at home or in a service flat (flat with domestic service)	Community	Nurse-led integrated care models	● Hospital admission ● ED visit	Critical low
Ekdahl et al. (2015) [38]	RCT, observational studies	6005 (17)	65 years or older; admitted to hospital with a complex condition; divided into frail and moderately frail group	Hospital	CGA-ward and CGA-consult	● Hospital readmission	Critical low
Ellis et al. (2017) [39]	RCT	13,766 (29)	65 years or older; admitted to hospital for acute care/ inpatient rehabilitation after acute admission	Hospital	CGA-ward and CGA-consult	● Hospital readmission ● LOS	Low
Ellis et al. (2011) [40]	RCT	10,315 (22)	65 years or older; admitted to emergency	Hospital	CGA-ward and CGA-consult	● Hospital readmission	Critical low
Facchinetti et al. (2020) [41]	RCT	8920 (30)	65 years or older; diagnosed with one or more chronic diseases; discharged home from hospital	Hospital	Continuity of care	● Hospital readmission	Critical low
Forster et al. (2008) [42]	RCT, quasi-RCT	3007 (13)	60 years or older; medical patients	Hospital	Medical day hospital	● Bed days	Critical low
Fox et al. (2012) [43]	RCT, quasi-experimental trial	6839 (13)	65 years or older; acutely ill or injured adults	Hospital	CGA-ward	● Hospital readmission ● LOS	Critical low
Hill-Taylor et al. (2016) [44]	RCT	1925 (4)	65 years or older	Hospital, LTCF	STOPP/START	● ED visits ● Drug use ● Primary care visit	Critical low
Lee et al. (2019) [45]	RCT, pre-post, retrospective, case-control	30,376 (50)	65 years or older; nursing home residents	LTCF	Pharmacists’ services in nursing home	● Hospital admission	Critical low
Lowthian et al. (2015) [46]	RCT, quasi-RCT, uncontrolled before/after, comparative controlled cohort, before/after observational	22,502 (9)	65 years or older; discharged home from ED	Hospital	ED-community transition strategies	● ED revisit ● Hospital admission	Critical low
Luker et al. (2019) [47]	RCT, cRCT	17,441 (31)	65 years or older, living in their own homes	Community	Community-based, aged-care interventions	● Hospital admission ● Hospital readmission ● Primary care visit	Critical low
Mayo-Wilson et al. (2014) [48]	RCT	28,642 (64)	65 years or older; community-dwelling adults without dementia	Community	Preventive home visit	● Hospital admission	Critical low
Poupard et al. (2019) [49]	RCT, cRCT	5468 (9)	65 years or older; community-dwelling adults	Community	Community-based case management	● Hospital admission ● ED visit ● LOS ● Bed days	Critical low
Spiers et al. (2019) [50]	Quasi-experimental, observational study	NR (12)	60 years and older, Living in a high-income country	Community	Social care supply	● Hospital admission ● LOS	Critical low
Rodakowski et al. (2017) [51]	RCT	4361 (13)	65 years and older; Discharge to a community setting with an informal caregiver	Hospital, LTCF	Caregiver integration during discharge planning	● Hospital readmission ● LOS	Low
Sadowski et al. (2020) [52]	RCT, nRCT, observational, chart review	20,228 (26)	Mean age > 80 years; multimorbid frail population in LTC	LTCF	Medication review by pharmacist in LTC	● Hospital admission	Critical low
Tecklenborg et al. (2020) [53]	RCT, case-control	2098 (7)	60 years and older; Receiving medical treatment in primary care	Primary care	Interventions to reduce ADEs in primary care	● Hospital admission ● ED visit	Critical low
Thillainadesan et al. (2020) [54]	RCT, prospective before-after	3026 (24)	Mean or median age 65 years and older; hospitalised under non-orthopaedic surgical teams for operative/nonoperative management	Hospital	Perioperative geriatric interventions	● Hospital readmission ● LOS	Critical low
Wallerstedt et al. (2014) [55]	RCT, nRCT	10,861 (12)	Mean age 78–86 years old; nursing home residents with drug treatment	LTCF	Medication reviews in nursing homes	● Hospital admission	Critical low
Weeks et al. (2018) [56]	RCT, comparable cohort/case-control	20,997 (23)	60 years and older; Community-dwelling with at least one medical diagnosis	Hospital, Community	Transitional care programs for community-dwelling older adults	● Hospital readmission ● LOS ● ED visit ● Primary care	Critical low
Wong et al. (2017) [57]	RCT	14,364 (22)	65 years and older; Living independently, with or without chronic diseases	Community	Community-based complex interventions	● Hospital admission	Low
Crespo-Rivas et al. (2021) [58]	RCT, nRCT, controlled before-after	NR (12)	60 years and older; residents of LTCFs	LTCF	Anti-microbial stewardship	● Hospital admission ● Drug use	Critical low
Williams et al. (2022) [59]	RCT, quasi-RCT, cRCT	1302 (5)	65 years and older; Admitted to the acute care setting for medical reasons	Hospital	Early supported discharge	● Hospital readmission ● LOS	Critical low
O’Shaughnessy et al. (2022) [60]	RCT, quasi-RCT, cRCT	7496 (11)	65 years and older; Admitted to an AGU with acute medical complaints	Hospital	CGA-ward	● Hospital readmission ● LOS	Critical low
Lin et al. (2022) [61]	RCT	1992 (10)	60 years and older; Preparing or recently discharged from hospital	Hospital, Community	Home-based exercise programmes	● Hospital readmission	Critical low
Van Grootven et al. (2017) [62]	RCT, nRCT, prospective before-after	3590 (12)	65 years and older; Hospitalised patients	Hospital	In-hospital geriatric co-management	● Hospital readmission ● LOS	Critical low
Li et al. (2022) [63]	RCT	11,693 (30)	65 years and older; Patients hospitalised for chronic disease > 1 day with aftercare needs	Hospitals, Primary care	Integrating primary healthcare in aftercare	● Hospital readmission ● LOS	Critical low
Kua et al. (2019) [64]	RCT	18,408 (41)	65 years and older; Living in a nursing home	LTCF	Deprescribing Interventions	● Hospital admission	Low
Birtwell et al. (2022) [65]	RCT, cRCT, prospective RCT, quasi-RCT, quasi-experimental, prospective pre-post, controlled pre-post, nRCT, randomised case-control, controlled trial	32,722 (15)	65 years and older; Living in LTCF	Hospital, LTCF	Transitional care programs for LTCF residents	● Hospital readmission ● ED revisit ● Hospital LOS ● ED LOS	Critical low
Tomlinson et al. (2020) [66]	RCT, cRCT	17,664 (24)	65 years and older; Preparing for hospital discharge or had a recent discharge (intervention provided within one month of discharge or on first post-discharge primary care visit).	Hospital, Community	Enhanced medication continuity	● Hospital readmission	Critical low
Lee et al. (2013) [67]	RCT, prospective cohort, pre-post, before-after, retrospective cohort	9679 (20)	65 years and older	Hospital, Community, LTCF	Geriatric patient care by pharmacist	● Hospital admission ● Hospital readmission ● LOS ● Drug use	Critical low

*RCT* Randomized Controlled Trials, *cRCT* cluster Randomized Controlled Trials, *nRCT* Non-randomised Controlled Trials, *CGA* Comprehensive Geriatric Assessment, *LOS* Length of Stay, *LTC* Long-term Care, *LTCF* Long-term Care Facility, *ED* Emergency Department, *ADE* Adverse Drug Event, *AGU* Acute geriatric unit, *NR* Not reported

### Characteristics and mapping of long-term care interventions to the healthy ageing Framework

Altogether, 37 LTC interventions were included in the analysis (Table 2). Although various interventions share similar names, they were implemented in distinct settings, yielded diverse outcomes, featured varying durations of outcomes follow-up, or engaged different providers, warranting separate descriptions. No overlap of intervention from the same meta-analyses was identified.

**Table 2:** Characteristics of LTC interventions

Author	WHO Healthy Ageing Domain	Subdomain		Intervention	Provider	Setting
Almutairi et al. (2020) [31]	Manage chronic condition	Medication appropriateness		**Medication optimisation in residential aged/continuing care** Educational, medication review, clinical decision support with technology, and multidisciplinary case-conferencing interventions aimed to reduce inappropriate medication prescription, which involves misprescribing, overprescribing, and underprescribing.	Pharmacists Physicians Nurses General practitioners Computer program Geriatrician Multidisciplinary team	LTCF
Beswick et al. (2010) [32]	Promote capacity enhancing	Community-based complex intervention		**Community-based multifactorial interventions** A preventive approach involves multifactorial assessment, active management, referrals, or recommendations. Multifactorial assessment may include physical and medical evaluation along with assessment of mental function, social condition, lifestyle, and home safety.	Social workers Nurses Physicians Physiotherapists Pharmacists	Community
Briggs et al. (2022) [33]	Early detection and control	Comprehensive Geriatric Assessment (CGA)		**CGA in a community setting** Assessment and holistic management plan for older persons, which leads to interventions in the setting of the participant’s home (domiciliary Comprehensive Geriatric Assessment (dCGA)) or in a community setting other than the participant’s home (community Comprehensive Geriatric Assessment (cCGA)).	Healthcare professional with gerontological expertise (geriatrician, specialist nurse or therapist)	Community
Conroy et al. (2011) [35]	Early detection and control	CGA		**CGA-post-hospital discharge model** Hospital-based assessment is followed by health services in the community, such as home-based physiotherapy and occupational therapy or referrals to community services or GPs.	Geriatricians Nurses Physiotherapists Occupational therapists	Hospital setting back into the community.
Cochrane et al. (2016) [34]	Compensate loss of capacity			**Time-limited home care reablement services**Intensive (multiple home visits), person-centred, goal-directed interventions to help older persons regain the ability to complete activities of daily living. This differs from the traditional home care service, which is usually time-limited (typically 6–12 weeks).	Occupational therapistsPhysiotherapists	Community
Deschodt et al. (2013) [36]	Early detection and control	CGA		**CGA-consult** Also known as the CGA team model. CGA is delivered by a mobile geriatric consultation team that is not in control of patient management and is only involved in evaluating, discussing, and recommending treatment plans for older patients hospitalised in the nongeriatric ward.	Multidisciplinary team: Geriatricians Nurses Social workers Occupational therapists Physiotherapists Dietitians Pharmacists	Hospital (nongeriatric ward)
Deschodt et al. (2020) [37]	Support capacity enhancing	Transitional care		**ED-community transition strategies** Nurse-led integrated care models for home-dwelling older persons where a nurse assesses the client’s needs and coordinates the care. The model involves a person-centred care approach by performing CGA or tailoring holistic assessment for the patient with a clear focus on continuity of care. It includes methods for improving patient independence, including informal caregivers in decision-making and medical review.	Nurses	Home or service flat (flat with domestic service)
Ekdahl et al. (2015) [38]	Early detection and control	CGA		**CGA-ward and CGA-consult**^a^ CGA-ward: an interdisciplinary team that assesses, plans, and takes full responsibility for all clinical decisions for older patients hospitalised in a geriatric ward. Covers both acute care and inpatient rehabilitation care programs. Interventions include assessments of physical and psychosocial function, medical care review, and discharge planning. CGA-consult is a mobile multidisciplinary team that assesses, discusses, and recommends a treatment plan for frail older inpatients in a non-geriatric ward. The interventions include a multidimensional evaluation that includes problem identification and recommendations, which will be consulted with the patient’s physician or included in the patient chart.	CGA-ward team: Geriatricians Nurses Therapists Psychologists Audiologists Dietician Social workers CGA-consult team: Geriatrician Nurses Therapists Social workers	Hospital
Ellis et al. (2017) [39]	Early detection and control	CGA		**CGA-ward and CGA-consult**^a^ Both types of CGA (CGA ward and consult) include the interventions of multidimensional assessment of medical, functional, mental, social, and environmental problems, multidisciplinary meetings, formulation of a plan of care which is patient-centred, delivery and review of said plan.	Geriatricians Healthcare assistants Nurses Therapists Pharmacists Dieticians Audiologists Psychologists Social workers	Hospital
Ellis et al. (2011) [40]	Early detection and control	CGA		**CGA-ward and CGA-consult**^a^ Both types of CGA (CGA ward and consult) include the interventions of multidimensional assessment of medical, functional, mental, social, and environmental problems, multidisciplinary meetings, formulation of a plan of care which is patient-centred, delivery and review of said plan.	Geriatricians Nurses Therapists Dieticians Psychologists Social workers	Hospital
Facchinetti et al. (2020) [41]	Support capacity enhancing	Continuity of care		**Continuity of care** Focus on connecting and coordinating patients and providers across time and settings. They are classified into informational, management, and relational continuity.	Nurses Physiotherapists Pharmacists Respiratory therapists Social worker Dietician	Hospital setting back into the community.
Forster et al. (2008) [42]	Manage chronic condition	Hospital care alternatives		**Medical day hospital** Day Hospital allows elderly patients to undergo comprehensive rehabilitation from a team of healthcare professionals in a healthcare setting. The intervention focuses on physical rehabilitation as a treatment goal.	Physiotherapists	Hospital (outpatient setting)
Fox et al. (2012) [43]	Early detection and control	CGA		**CGA-ward**^a^ CGA ward, also known as acute geriatric unit care, includes at least one of the Acute Care for Elders (ACE) model components, such as patient-centred care that has assessments and protocols to prevent declines in various aspects of a patient’s well-being, frequent medical review, early rehabilitation, and prepared environment, which includes environmental modifications to aid physical and cognitive functioning	Geriatrician Medical director Orthopaedic surgeon Nurses Social workers Physiotherapists Occupational therapists Dietitian	Hospital (acute geriatric ward)
Hill-Taylor et al. (2016) [44]	Manage chronic condition	Medication appropriateness		**STOPP/START** On admission to the hospital, patients received screening with STOPP and/or START criteria to identify prescribing appropriateness. One paper had the nursing home physician receive education regarding STOPP/START to be applied for the resident’s medication screening.	Pharmacists Nursing home physicians Geriatricians	Hospital, LTCF
Lee et al. (2019) [67]	Manage chronic condition	Medication appropriateness		**Pharmacists’ services in nursing home** The services include clinical medication review, staff education, and multidisciplinary team meetings. Intervention for medication review would address various medication-related aspects. Staff education might involve face-to-face education with relevant professionals. Another intervention would include team meetings, which would make decisions regarding the treatment of the recommendation and its execution.	Pharmacists	LTCF
Lowthian et al. (2015) [46]	Support capacity enhancing	Transitional care		**ED-community transition strategies** Any interventions that have assessment during the discharge of patients from ED to formulate a discharge care plan and arrangement of community service provision. The evaluation might include comprehensive geriatric nurse assessment with/without other screening tools to identify high-risk patients or by health visitor home visit within 24 h of ED discharge.	Nurses General practitioners Social workers	Hospital setting (discharge from ED) back into the community
Luker et al. (2019) [47]	Promote capacity enhancing	Community-based complex intervention		**Community-based, aged-care interventions** Interventions that enable older persons to continue living in their own homes as they grow old. Include centre-based wellness programs, re-enablement or restorative home care, case management, and consumer-directed care where the client controls services of interest.	Multidisciplinary team General Practitioners Nurses Pharmacists Physiotherapists Social workers	Hospital, Community
Mayo-Wilson et al. (2014) [48]	Promote capacity enhancing	Home visit		**Preventive home visit** Visitation would involve providing information, investigating untreated problems, supporting medication compliance, or referring to health services.	Nurses Health visitors Physiotherapists Social workers Occupational therapists	Hospital, Community
Poupard et al. (2019) [49]	Manage chronic condition	Community-based case management		**Community-based case management** Appointment of case manager and home visits. It comprises comprehensive assessment, medication review, individualised care plan, monitoring, care coordination, self-management strategies, fall prevention, caregiver support, and ongoing referrals and medical appointments.	Multidisciplinary team Case manager: Social workers, nurses, allied health professionals, physiotherapists, physician	Community
Rodakowski et al. (2017) [51]	Support capacity enhancing	Coordinated/Integrated care		**Caregiver integration during discharge planning** The components included linking caregivers to external or community resources, a written care plan, caregiver assessment, medication reconciliation, live or video demonstration, and a teach-back technique (caregiver/patient demonstration to the interventionist) for the care tasks.	Nurses Geriatricians Multidisciplinary team (not described) Discharge coordinator/case manager Research assistants	Hospital or skilled nursing facility setting to community
Sadowski et al. (2020) [52]	Manage chronic condition	Medication appropriateness		**Medication review by the pharmacist in LTC** The intervention harnesses pharmacists’ expertise to address the complexity of medication regimens (MRC) resulting from increased polypharmacy and prevent associated issues like adverse drug events (ADEs) and ED visits. It involves medication review, documentation, case conferences, and educational activities as key components.	Pharmacist	LTCF
Tecklenborg et al. (2020) [53]	Manage chronic condition	Medication appropriateness		**Interventions to reduce the incidence of ADEs** Any intervention aimed at reducing harmful, unpremeditated effects of medication usage. This includes prescription reviews using established prescribing indicators such as STOPP/START, Beers Criteria, and MAI. It also involves medication reviews such as the focussed medication review, number of drugs and risk indicators, computer-based assessment of potential drug interaction, and educational training for nursing staff.	Pharmacist Physician Research team Nurses	Primary care
Thillainadesan et al. (2020) [54]	Prevent chronic condition	Perioperative geriatric management		**Perioperative geriatric interventions** The interventions were preoperative, postoperative, or both to enhance clinical outcomes in older surgical patients. This includes multicomponent inpatient geriatric programs, preoperative cognitive training and exercise programs primarily based on modifying the HELP program, the preoperative CGA and management, and prehabilitation.	Geriatrician Surgeon General Physician Nurse Physiotherapist Occupational therapist Social worker Psychologist Dietician Other therapist	Hospital (non-orthopaedic surgical teams for operative or nonoperative management)
Wallerstedt et al. (2014) [55]	Manage chronic condition	Medication appropriateness		**Medication reviews in nursing homes** The intervention comprises systematic assessments aimed at evaluating and optimising medication prescriptions. This includes medication reconciliation to ensure accurate prescriptions and assessing medication appropriateness using established indicators such as the Medication Appropriateness Index (MAI) or Beers criteria.	Multiprofessional team Pharmacist Physicians Geriatricians Geriatric nurse General practitioner	LTCF
Weeks et al. (2018) [56]	Support capacity enhancing	Transitional care		**Transitional care programs for community-dwelling older persons** The intervention aimed to coordinate and ensure the continuity of healthcare for older persons as they transition between different healthcare settings. This included personalised activities such as coordinating care among various providers and settings, providing access to healthcare and community services, and monitoring health and medication management. The intervention was delivered through home/community visits, phone calls, nurse coaching sessions upon discharge, and symptom monitoring using technology.	Nurse Social worker Interdisciplinary/interprofessional team Transition coach Community-health worker Patient navigator	Hospital setting back into the community.
Wong et al. (2017) [57]	Promote capacity enhancing	Community-based self-care		**Community-based complex interventions** This multifaceted program is designed to promote and support self-care among older persons living independently in the community. The intervention includes educational initiatives, personalised care planning, regular assessments, healthcare professional guidance, and peer support networks. It was delivered to the community through home or community visits, telephone follow-ups, and group training.	Nurse Geriatrician Physician Dietician Physiotherapist Occupational therapist Clinical social worker Care manager General practitioner	Community
Crespo-Rivas et al. (2021) [58]	Manage chronic condition	Medication appropriateness		**Antimicrobial stewardship** The intervention involves back-end and front-end strategies to control antimicrobial consumption (overuse/misuse) and prevent the risk of adverse effects on the older residents of the LTCFs. The multifaceted ASPs include educational, tailored intervention and strategies, audit and feedback, promotion of clinical practice guidelines, incorporation of local drug therapeutic committee guidelines, personalised advice from infectious disease teams for antibiotic use, and patient-mediated interventions.	Physician Nurses Pharmacists Residents Family member	LTCF
Williams et al. (2022) [59]	Support capacity enhancing	Transitional care		**Early supported discharge** The interventions involve inter/multidisciplinary teams that aim to link acute and community care. Patients are discharged, and care extends to their homes with home rehabilitation, daily nursing reviews, and up to 24-hour in-home caregivers. Comprehensive geriatric assessments are performed both in the hospital and during post-discharge follow-up. Goal setting and care planning were also included.	Multidisciplinary team (varies) Medical doctors General practitioners Case manager (nurse) Physiotherapist Occupational therapist Social worker	Hospital setting back into the community.
O’Shaughnessy et al. (2022) [60]	Early detection control	CGA		**CGA-ward**^a^ The care model aimed to prevent functional decline and related complications in older patients admitted to the acute care setting. The intervention component includes clinical leadership, structured assessment, multidisciplinary team meetings, goal setting, involving patients and carers in goal setting, outpatient follow-up, ward environment, adequate time, speciality knowledge, experience, and competence, and tailoring treatment plans to the individual.	Trained nurse Physician Social worker Occupational therapist Physical therapist Pharmacist Geriatrician Nutritionist Dietitian	Hospital (Acute geriatric unit care)
Lin et al. (2022) [61]	Promote capacity enhancing		**Home-based exercise programmes** The intervention aimed to enhance physical activity and improve older patients’ function and quality of life upon discharge. The components included tailored exercises such as fall prevention exercises, strengthening exercises, balance exercises, functional exercises, mobility training, and education advice.	Physiotherapist Healthcare professional (exercise physiologist, exercise scientist)	Hospital setting back into the community	
Van Grootven et al. (2017) [62]	Support capacity enhancing	Coordinated/Integrated care		**Inhospital geriatric comanagement** The intervention comprised early rehabilitation, medical care review, discharge planning and patient-centred care by the treating physician and a geriatrician. It aimed to improve the quality of care for older frail patients hospitalised in non-geriatric wards.	Physical therapist Social worker Geriatric nurses Occupational therapist Internal medicine resident	Hospital (Non-geriatric ward)
Li et al. (2022) [63]	Support capacity enhancing	Continuity of care		**Integrating primary healthcare in aftercare** The interventions aimed to enhance the continuity of care between hospitals and primary healthcare facilities. These interventions included longitudinal continuity, information continuity, communication continuity and management continuity, primarily through care coordination between hospital nurses and healthcare providers.	Nurse Physician Therapist Pharmacist General practitioner Geriatrician social worker Community health care worker	Hospitals, primary care
Kua et al. (2019) [64]	Manage chronic condition	Medication appropriateness		**Deprescribing Interventions** The intervention includes drug discontinuation, medication review and educational training for nursing home staff to reduce potentially inappropriate medication (PIM) use due to polypharmacy and prevent adverse clinical events or outcomes for nursing home residents.	Physician Pharmacist Multidisciplinary teams (Physician, pharmacist, nurse, occupational therapist, and psychologist)	LTCF
Birtwell et al. (2022) [65]	Support capacity enhancing	Transitional care		**Transitional care programs for LTCF residents** The intervention aimed to enhance the quality of care for older persons residing in LTCFs during transitions between care settings. This includes discharge planning, post-discharge communication and support, implementing innovative care models and pathways, medication review, staff training and education within LTCFs or hospitals.	Nurse Pharmacist Physician Healthcare/community/primary care practitioner who received information and support as part of the intervention	Hospital setting back into the LTCF or vice versa.
Tomlinson et al. (2020) [66]	Support capacity enhancing	Continuity of care		**Enhanced medication continuity** Interventions that bridge care transitions following patient discharge to prevent medication-related problems and support medication continuity include home visits, telephone follow-up, self-management, medication reconciliation activities, and electronic intervention (RightRx).	Pharmacist Geriatrician Nurse Multidisciplinary team (not mentioned) RightRx (electronic application)	Hospital setting back into the community
Lee et al. (2013) [45]	Manage chronic condition	Medication appropriateness		**Geriatric patient care by pharmacist** Patient-level pharmacist intervention in geriatric care involves educational, behavioural and technical intervention to optimise medication use while minimising potential side effects in older patients.	Pharmacist	Hospital, LTCF, Community
Spiers et al. (2019) [50]	Promote capacity enhancing	Social care		**Social care supplies** Social care is the availability and provision of services within a community or society that aim to support older patients with various aspects of their daily lives, typically in their homes or care homes.	State government or individual	Community

*CGA* Comprehensive Geriatric Assessment, *LTCF* Long-term Care Facility, *ED* Emergency Department, *ADE* Adverse Drug Event, *GP* General Practitioners, *LTCF* Long-term Care Facilities

^a^Studies with similar interventions but either applied at different settings, had different outcomes, or had different duration of outcomes follow-up

Most interventions involved multidisciplinary teams or coordination, with only six interventions among a single healthcare professional [37, 42, 45, 50, 52, 67]. The most common settings were community-based or involved transfer back to the community following discharge from the hospital, including the older person’s home, with 17 interventions. Nine interventions were set in long-term care institutions [31, 44, 45, 51, 52, 55, 58, 64, 65], with the remaining in hospitals or in mixed settings. The most common type of intervention was the Comprehensive Geriatric Assessment (CGA) (*n* = 8, 23.5%). However, the intervention was applied across various settings, delivered by different teams of healthcare professionals, and had different follow-up durations. Mapping to the WHO Healthy Ageing Framework revealed that 11 interventions focused on managing chronic conditions [31, 42, 44, 45, 49, 52, 53, 55, 58, 64, 67], ten supported capacity enhancement [37, 41, 46, 51, 56, 59, 62, 63, 65, 66], eight were on early detection and control [33, 35, 36, 38–40, 43, 60], six were to promote capacity enhancement [32, 47, 48, 50, 57, 61], and one each for prevention of chronic conditions [54] and compensation of capacity [34].

### Associations between long-term care interventions with healthcare utilisations among older persons

One hundred and twelve associations were reported between LTC interventions and healthcare utilisations, mostly on hospital utilisation (*n* = 86, 76.8%). Seventeen associations were reported on ED utilisation, six on medication utilisation and three on primary care service utilisation (Additional file 6). Twenty-two out of the 112 associations (19.6%) were statistically significant (Table 3). The GRADE reporting for all associations is reported in Additional File 7.

**Table 3:** Summary of Significant Associations of Long-term Care Interventions with Healthcare Utilisation

Interventions	Study design	No. of studies	*n*	Follow-up (months)	Heterogeneity (I^2^), %	Effect size, Random (95% CI)	*p*-value	GRADE rating
**HOSPITAL UTILISATION**								
**Hospital admission**								
Deprescribing Interventions Kua et al. (2019) [64]	RCT	1	95	12	-	**OR: 0.40 (0.17, 0.92)**	**0.031**	Very low
Community-based, aged-care interventions Luker et al. (2019) [47]	RCT	1	739	18	-	**OR: 0.67 (0.50, 0.89)**	**0.006**	Very low
	RCT	1	294	24	-	**MD: -0.38 (-0.69, -0.07)**	**0.016**	Very low
Preventive home visit Mayo-Wilson et al. (2014) [48]	RCT	7	2155	7–12	0	**OR: 0.73 (0.59, 0.91)**	**0.005**	**Moderate**
CGA in a community setting Briggs et al. (2022) [33]	RCT	2	583	13–24	0	**OR: 0.57 (0.41, 0.80)**	**0.001**	**Low**
Medication review by pharmacist in LTCF Sadowski et al. (2020) [52]	RCT	2	169	12	10.4	**OR: 0.16 (0.03, 0.73)**	**0.019**	Very low
**Hospital readmission**								
Caregiver integration during discharge planning Rodakowski et al. (2017) [51]	RCT	13	5734	1–6	32.0	**OR: 0.68 (0.57, 0.81)**	**< 0.001**	**High**
Transitional care programs for community-dwelling older persons Weeks et al. (2018) [56]	RCT	10	7751	1–6	77.4	**OR: 0.79 (0.62, 1.00)**	**0.048**	Very low
	OBS	2	2537	1–6	22.6	**OR: 0.54 (0.38, 0.76)**	**0.000**	Very low
Transitional care programs for LTCF residents Birtwell et al. (2022) [65]	Mix	11	NR	1–6	40.0	OR: 1.48 (1.01, 2.17)*	Reported significant*	Very low
Integrating primary healthcare in aftercare Ran Li et al. (2022) [63]	RCT	22	3990	1–6	71.4	**OR: 0.60 (0.49, 0.74)**	**< 0.001**	**Low**
Continuity of care Facchinetti et al. (2020) [41]	RCT	21	6407	1–6	64.0	**OR: 0.80 (0.66, 0.97)**	**0.026**	Very low
	RCT	12	2066	7–12	30.8	**OR: 0.76 (0.61, 0.95)**	**0.018**	**Moderate**
Community-based, aged-care interventions Luker et al. (2019) [47]	RCT	1	412	6	-	RR: 1.30 (1.07, 1.58)*	**0.009**	**Low**
**Length of stay**								
Early supported discharge William et al. (2022) [59]	RCT	4	1023	Hosp	90.1	**MD: -6.04 (-9.76, -2.32)**	**0.001**	Very Low
Perioperative geriatric interventions Thillainadesan et al. (2020) [54]	RCT	8	1179	pre/post operative	0	**MD: -1.57 (-2.21, -0.93)**	**< 0.001**	**Moderate**
CGA-ward Fox et al. (2012) [43]	RCT	7	5128	Hosp, 3	51.7	**MD: -0.62 (-1.24, -0.01)**	**0.047**	Very low
**ED UTILISATION**								
**ED visit**								
Community-based case management Poupard et al. (2019) [49]	RCT	1	92	12	-	**MD: -0.50 (-0.96, -0.04)**	**0.034**	Very low
CGA in a community setting Briggs et al. (2022) [33]	RCT	1	199	12	-	**OR: 0.32 (0.12, 0.84)**	**0.02**	**Low**
**Length of stay**								
Transitional care programs for LTCF residents Birtwell et al. (2022) [65]	MIX	3	679	NR	99	SMD: -3.51 (-3.61, -2.39)*	Reported significant*	Very low
**MEDICATION UTILISATION**								
**Drug Use**								
Anti-microbial stewardship Crespo-Rivas et al. (2021) [58]	RCT	3	84	12	71	MD: -0.47 (-0.87, -0.07)*	**0.02**	Very low
**PRIMARY CARE UTILISATION**								
**Primary care visit**								
Community-based, aged-care interventions Luker et al. (2019) [47]	RCT	1	NR	NR	-	RR: 1.43 (1.14 to 1.80)*	**0.002**	Very low

*RCT* randomised controlled trial, *OBS* observational study, *MIX* mixed study design, *LTCF* long-term care facility, *ED* emergency department, *CGA* comprehensive geriatric assessment, *Hosp*: hospitalisation, *MD* mean difference, *SMD* standardised mean difference, *OR* odd ratio, *RR* risk ratio, *95% CI* 95% Confidence Interval, *n* = total number of participants in trials, *sig*. significant, *NR* not reported, *GRADE* Grading of Recommendations, Assessment, Development and Evaluations

*Value taken from meta-analysis paper due to insufficient data for reanalysis

### Hospital utilisation

Altogether, 35 associations discussed hospital admission (Additional file 6). Six associations (17.1%) reflected a significant reduction in the risk of hospital admission among older persons. The associations mapped to five interventions: Deprescribing interventions [64], community-based aged care [47], preventive home visits [48], CGA implemented in a community setting [33], and medication review by pharmacists in Long-term Care Facility (LTCF) [52]. Three of the five significant interventions were implemented in the community-based setting (community-based aged care [47], preventive home visits [48], and CGA implemented in a community setting [33]). Among these five interventions, only preventive home visits at 7–12 months follow-up (OR: 0.73, 95% CI: 0.59, 0.91, *p* = 0.005) received a moderate GRADE quality of evidence rating [48], with all other interventions rated either low or very low quality.

For hospital readmission, there were 32 associations (Additional file 6). Eight associations (25%) from six interventions were significantly associated with hospital readmission. All six interventions involved implementation in a community setting or a transfer back into the community following discharge from the hospital [41, 47, 51, 56, 63, 65]. Six associations were found to reduce hospital readmission, with only one intervention, caregiver integration during discharge planning at 1–6 months follow-up (OR: 0.68, 95% CI: 0.57, 0.81, *p* < 0.001), had high-quality evidence [51]. This intervention included 13 studies with a low heterogeneity. Another intervention found to have a moderate quality of evidence in reducing hospital readmission was continuity of care at a 7–12 months follow-up (OR: 0.76, 95% CI: 0.61, 0.95, *p* = 0.018) [41]. The other two interventions representing four associations with low and very low-quality evidence were integrating primary healthcare in aftercare [63] and transitional care programs for community-dwelling older persons [56]. The remaining two interventions increased the odds or risk of hospital readmissions among older persons (transitional care programs for long-term care facility residents [65] and community-based aged-care interventions [47]). However, both interventions received low and very low-quality evidence.

There were 17 associations regarding the outcome of length of stay (Additional file 6). Three associations (17.6%) from three interventions demonstrated significant reductions in the length of hospital stay [43, 54, 59]. All interventions were implemented in a hospital setting. Perioperative geriatric interventions at a 12-month follow-up, which involved twelve studies, were the only intervention with a moderate quality of evidence (MD: -1.50, 95% CI: -2.24, -0.76, *p* < 0.001) and low heterogeneity (32.7%) [54]. The remaining two interventions, early support discharge [59] and CGA, were implemented in the ward and had low or very low-quality evidence and high or moderate heterogeneity.

### Utilisation of emergency department

ED utilisation was found to have 17 associations (Additional file 6). Three associations (17.6%) from three interventions significantly reduced ED utilisation. Two were on ED visits (community-based case management [49] and CGA implemented in a community setting [33]), and one was on the length of ED stay (transitional care programs for long-term care facility residents [65]). However, all three interventions were found to have either low or very low-quality evidence.

### Utilisation of medications and prescriptions

The outcome of drug use had six associations (Additional file 6). Only one association (16.7%) from an intervention, anti-microbial stewardship at a 12-month follow-up, significantly reduced the number of drugs used among older persons (MD: -0.47, 95% CI: -0.87, -0.07, *p* = 0.02) [58]. However, this association was graded as having very low quality and high heterogeneity.

#### Utilisation of primary care

Three associations were found for the outcome of primary care visits (Additional file 6). One association (33.3%) from an intervention was statistically significant. A community-based aged care intervention increased the number of visits (RR: 1.43, 95% CI: 1.11, 1.18, p: 0.002) [47]. The quality of evidence was found to be very low.

### Sensitivity analysis

The re-analysis of associations with high heterogeneity using a fixed-effect model did not significantly alter the associations between the intervention and the outcome measured.

## Discussion

Thirty-seven meta-analyses were included in the study, comprising 112 associations between various LTC interventions and healthcare utilisations. Four of the 22 statistically significant associations were supported by suggestive or convincing evidence and remarked as either high or moderate quality of evidence. These associations include four different LTC interventions: preventive home visits were found to reduce hospital admission [48], caregiver integration during discharge planning [51], and continuity of care [41], reduced hospital readmission, and perioperative geriatric interventions [54] reduced the length of hospital stay. There was no convincing evidence on the association between LTC and ED, medication and primary care utilisation.

Mapping the LTC interventions to the WHO Healthy Ageing Framework revealed that the most extensive domain explored was managing chronic conditions (11 out of 37 interventions), followed by support of capacity enhancement (9 out of 37 interventions). These findings fit well with the aims of the healthy ageing framework, whereby both domains were crucial in preventing substantial loss of capacity among older persons [7]. However, it represents opportunities or a need to explore services in other domains, facilitating the evidence-based implementation of more comprehensive LTC services. This is essential for supporting health systems in meeting the evolving needs of the ageing population, ensuring that older persons receive high-quality and coordinated care for their well-being. Most interventions were found to be implemented in a community setting or involved a transfer back into the community following discharge from the hospital. This finding aligns with other evidence emphasising that while LTC services can be implemented in various settings, a community-based approach offers the most benefit to older persons [7, 19, 68]. However, this finding could also be due to the exclusion of interventions among disease-specific conditions, which may have been more extensively implemented in hospital or institutional-based settings. Therefore, future comparisons and discussions should consider the contextual factors of LTC implementations, including the specific settings in which they occur.

Most interventions involved multidisciplinary teams of various health, social care, and community-based providers, supporting findings and recommendations elsewhere [7, 20]. Indeed, the involvement of relevant providers in caring for and providing LTC services for older persons is essential in ensuring that all their needs are adequately assessed and addressed in an integrated and coordinated approach [12, 68]. CGA emerged as the most common intervention recurring across all meta-analyses. It signifies the importance of a thorough clinical and psychological evaluation and the presence of support evaluation in delivering care to older persons [69]. However, since this review identified interventions labelled as ‘long-term care’, it could also suggest that CGA was among the most developed LTC interventions that have been assessed and evaluated across multitudes of different outcomes, including healthcare utilisations, which highlights future research opportunities for evaluating other LTC interventions that were less explored.

This review found suggestive evidence that preventive home visits [48] reduced the likelihood of hospital admission among older persons. The service was provided by a multidisciplinary team offering comprehensive care, including assessment of health and support needs, referral to relevant care providers, medication review, and rehabilitation at the older person’s home [48]. The approach ensures early detection of diseases and conditions, improves access to care, and offers a large spectrum of services that are otherwise not provided during routine care [70]. The finding reciprocates a recent umbrella review reporting that home visits were favourable in reducing hospital admission frequency [71]. As debated in the review, the definitions and components of what constituted ‘home visits’ vary across studies and warrant further evaluation. Nevertheless, the heterogeneity for this intervention was found to be low in this study [72].

Caregiver integration during discharge planning had convincing evidence in reducing hospital readmission [51]. The service included linking caregivers to external or community resources, preparing written care plans, performing caregiver assessment, medication reconciliation, and iterative teaching sessions in providing care, all planned and executed during the discharge process of older persons from the hospital. Discharge planning implies a comprehensive plan was prepared based on the anticipated healthcare needs of the older persons [73]. Including caregivers in the discharge planning enhanced the care by delegating part of the responsibilities to the person managing the patient [74]. Similarly, continuity of care upon discharge from the hospital was found to have suggestive evidence in reducing hospital readmission [41]. Early hospital readmissions are often due to insufficient recognition of a patient’s needs, leading to unaddressed issues and poor management at home [75]. Ensuring that care is continued primarily through care coordination between hospital and primary healthcare providers means the underlying disease that caused the earlier hospital admission is appropriately managed. A recurrent episode requiring further hospitalisation (readmission) is thus prevented [41]. The overarching idea was that to reduce hospital readmission, there was a need for a comprehensive assessment and identification of an older person’s health and other requirements within the hospital setting before discharge. This process involves crafting a detailed, coordinated care plan that includes caregivers and other healthcare providers, ensuring a smooth transition and effective ongoing care management post-discharge.

Perioperative geriatric interventions, defined as any program aiming to enhance clinical outcomes of older persons having surgeries performed, was the only intervention with suggestive evidence of reducing the length of stay in hospital [54]. The interventions mitigate the increased risk associated with surgeries by customising care to the specific needs of the patients, potentially preventing functional decline and related complications [54]. No convincing LTC intervention was found to influence ED, drug use, and primary care utilisation. This could be due to the limited number of studies that evaluated these outcomes, warranting future research.

### Implications for practice and future research

This scoping review offers an extensive summary across meta-analyses on existing LTC interventions that impacted healthcare utilisations. Since the scope of this study covers the general older population, the findings may be beneficial for policymakers looking to implement LTC interventions at a macro rather than disease-specific level. The review found a few effective LTC interventions in reducing healthcare utilisations. Nevertheless, from the health systems point of view, it provides insights for potential interventions that could alleviate the strain on healthcare systems, exacerbated by the increasing prevalence of non-communicable diseases (NCDs) and age-related functional disabilities [76]. It also means future research may be directed towards exploring the impact of more and newer LTC interventions towards healthcare utilisations. The limited number of existing studies assessing the impact of LTC interventions on healthcare utilisation may partly explain the little evidence found through this study. Still, it also suggests that LTC interventions could have broader positive effects on various other outcomes, including individual well-being, mortality, clinical outcomes, and functional limitations, which were not within the scope of the current study. Despite the small number of effective LTC interventions found, the overall picture suggests that LTC interventions need to move away from hospital or institution-based implementations to the community or older persons’ homes. Such a move offers integrated, person-centred care at the place most comfortable for the older person, increasing adherence to care [77]. Comprehensive assessment identifying all aspects of the needs of older persons while being hospitalised, coupled with a properly documented detailed discharge plan, which includes roles of carers and other professionals that will continue the care upon discharge, is crucial in ensuring older persons’ well-being, hence impacting the subsequent healthcare utilisation.

### Limitations

This scoping review has several limitations. The search strategy relied on interventions labelled ‘long-term care’ or other terms referring to LTC interventions. While the keywords postulated in the study covered as comprehensive LTC interventions as possible, more interventions may have served LTC functions but were not labelled as such. While the study covers multiple outcomes related to healthcare utilisations, more outcomes existed that were not included in the current review. For example, preventable hospitalisation, preventable ED visits, and time to hospitalisation were among various related outcomes not included in the present review. While the inclusion of meta-analysis in our review offers a comprehensive overview of the outcomes’ direction and strength across different interventions, it is essential to acknowledge the diversity in implementation, contextual backgrounds, and settings of various other interventions, making it impossible for studies to pool and analyse all existing interventions. As a result, while our current review encompasses broad LTC interventions for older persons analysed within a systematic review with meta-analysis, it is essential to recognise that a wealth of additional evidence available could offer further insights into practical strategies for reducing healthcare utilisation among this population. The review also did not directly assess the quality of individual primary studies included in each meta-analysis but instead relied on the assessment reported by the authors. A further limitation was that we did not perform subgroup analysis (for example, by age groups, sex, and location where the intervention was delivered) due to the lack of data for grading the quality evidence for most interventions.

## Conclusion

The findings of this study suggest LTC interventions could benefit from transitioning to a community-based setting, involving a multidisciplinary team including the carers that offer a large spectrum of services fulfilling various needs of older persons, incorporate comprehensive and holistic assessment plan, and include a detailed discharge plan that ensures integrated, coordinated and continuous care is achieved. However, the current evidence pertains to hospital utilisation, with more research needed to identify interventions impacting other healthcare utilisations. Nevertheless, the present findings offer insights into effective LTC interventions that may be considered for implementation by policymakers at a macro level.

### Supplementary Information

Additional file 1. Search strategy 

Additional file 2. Template for extraction record

Additional file 3. Flowchart for reanalysing extracted values

Additional file 4. Excluded Articles

Additional file 5. AMSTAR-2 assessment of all included studies

Additional file 6. Summary of associations between long-term care interventions with hospital utilisation among older persons

Additional file 7. GRADE assessment for significant associations

Additional file 8. PRIOR Checklist

Additional file 9. Preferred Reporting Items for Systematic reviews and Meta-Analyses extension for Scoping Reviews (PRISMA-ScR) Checklist

## Data Availability

The data analysed for this review is part of the ‘Simulation of Long-Term Care for Elderly in Malaysia’ (MyLTC) study and belongs to the Ministry of Health Malaysia. Requests for the data can be obtained from the Principal Investigator, Dr Fun Weng Hong, through email: fun.wh@moh.gov.my with permission from the Director-General of Health, Malaysia.

## Abbreviations

AMSTAR-2: A Measurement Tool to Assess Systematic Reviews-2
CGA: Comprehensive Geriatric Assessment
CI: Confidence interval
ED: Emergency department
GRADE: Grading of Recommendations, Assessment, Development, and Evaluations
IQR: Interquartile range
LMICs: Low- and middle-income countries
LTC: Long-term care
LTCF: Long-term Care Facility
MD: Mean difference
MeSH: Medical Subject Headings
MREC: Medical Research and Ethics Committee
NCDs: Non-communicable diseases
NR: Not reported
OR: Odds ratio
OSF: Open Science Framework
PRISMA: Preferred Reporting Items for Systematic Review and Meta-analyses
RCTs: Randomised Controlled Trials
RR: Risk ratio
WHO: World Health Organization
