# Supplementary material for: The impact of long-term care interventions on healthcare utilisation among older persons: a scoping review of reviews
Source: BMC Geriatr. 2024 Jun 3;24:484. doi: 10.1186/s12877-024-05097-9 (PMC11145838; doi:10.1186/s12877-024-05097-9)
Supplement: Supplementary file 1 — Additional file 1. Search strategy [file 12877_2024_5097_MOESM1_ESM.docx]

**Additional file 1: Search strategy**

| **#** | **Searches Terms** |
| --- | --- |
| **1** | Long-Term Care; Insurance, Long-Term Care/ Long Term Care; Long-Term Care; Long-Term Care; Model; Care; Aged Care |
| **2** | Home Care Agencies; Home Health Aides; Home Care Services; Social Support; Assisted Living Facilities; Homes for The Aged; Nursing Homes; Patient Care/ Home Care; Formal Care; Home Service*; Social Care; Home and Community-Based Services; Community Care; Informal Care |
| **3** | 1 or 2 |
| **4** | Aged; Aged, 60 and over; Frail Elderly; Geriatrics/ Elderly; Elderlies; Elder; Old age; Senior; an older person |
| **5** | 3 and 4 |
| **6** | Hospitalisation; Length of Stay; Patient Readmission; Clinical Laboratory Services; Medication Reconciliation; Emergency Care Visit; Emergency Department Visit; Emergency Department Revisit; Medication; Medication Utilisation; Pharmacy Related Practice; Primary Care; Primary Care Visit; Outpatient; Specialist Clinic; Hospital Readmission; Hospital Bed Day; Bed Day; Health Care Service; Health Service; Laboratory Service; Day Care; Ambulatory Care |
| **7** | 5 and 6 |
| **8** | Systematic review, meta-analysis, meta-analyses |
| **9** | 7 and 8 |

**PubMed**

1. “Long-Term care” [MeSH Terms] OR “Insurance, Long-Term Care” [MeSH Terms] OR "long term care" OR "long-term care" OR "longterm care" OR "aged care"
2. “Home Care Agencies” [MeSH Terms] OR “Home Health Aides” [MeSH Terms] OR “Home Care Services” [MeSH Terms] OR “Social support” [MeSH Terms] OR “Assisted Living Facilities” [MeSH Terms] OR “Homes for the Aged” [MeSH Terms] OR “Nursing Homes” [MeSH Terms] OR "home care" OR "formal care" OR "home service*" OR "social care" OR "home and community based services" OR "community care" OR "informal care" OR "home care agencies" OR "home care agency" OR "home health aides" OR "home care service*" OR "social support" OR "assisted living facilities" OR "homes for the aged" OR "nursing home*"
3. 1 OR 2
4. “Aged” [MeSH Terms] OR “Aged, 60 and over” [MeSH Terms] OR “Frail Elderly” [MeSH Terms] OR “Geriatrics” [MeSH Terms] OR “elderly” OR "elder person" OR "old age" OR “senior” OR "older person" OR aged OR "frail elderly" OR "old person" OR "geriatric*" OR "frail elderly"
5. 3 AND 4
6. “Hospitalization” [MeSH Terms] OR “Hospitalization” OR “Hospitalisation” OR “Hospitalisation” [MeSH Terms] OR “length of stay” OR “Length of Stay” [Mesh Terms] OR “patient readmission” OR “Patient Readmission” [Mesh Terms] OR "Clinical Laboratory Services" [Mesh] OR “Clinical Laboratory Services” OR "Medication Reconciliation" [Mesh] OR “Medication Reconciliation” OR “emergency care visit*” OR “emergency department visit*” OR “emergency department revisit*” OR “emergency care revisit*” OR “ED visit*” OR “ED revisit*” OR “medication” OR “medication utilisation” OR “pharmacy related practice*” OR “pharmacy practice*” OR “primary care” OR “primary care” [MeSH Terms] OR “primary care visit” OR “outpatient*” OR “Outpatients” [Mesh] OR “outpatient visit*” OR “specialist clinic” OR “specialist care” OR “specialist care visit*” OR “hospital admission” OR “hospital readmission” OR “hospital bed day” OR “bed day” OR “health care service” OR “health service” OR “Health Services” [Mesh] OR “laboratory service*” OR “day care” OR “ambulatory care” OR “Ambulatory Care” [Mesh] OR “Emergency department” OR “emergency care”
7. 5 AND 6
8. “Systematic review” [Title/Abstract] OR "meta-analysis" [Title/Abstract] OR "meta-analyses" [Title/Abstract]
9. 7 AND 8
10. Limiters - Full Text; Publication Year: 2010-2022; Human; Language: English

**EBSCO CINAHL Plus**

1. (mh “Long-Term care”) OR (mh “Insurance, Long-Term Care”) OR long term care OR longterm care OR aged care
2. [mh “Home Care Agencies”] OR [mh “Home Health Aides”] OR [mh “Home Care Services”] OR [mh “Social support”] OR [mh “Assisted Living Facilities”] OR [mh “Homes for the Aged”] OR [mh “Nursing Homes”] OR patient care OR home care OR formal care OR home service OR social care OR home and community-based services OR community care OR informal care OR home care agencies OR home health aides OR home care service OR social support OR assisted living facilities OR homes for the aged OR nursing home
3. 1 OR 2
4. [mh “Aged”] OR [mh “Aged, 60 and over”] OR [mh “Frail Elderly”] OR [mh “Geriatrics”] OR elderly OR elder person OR old age OR senior OR older person OR aged OR old person OR frail elderly OR geriatric
5. 3 AND 4
6. [mh “Hospitalization”] OR [mh “Hospitalisation”] OR [mh “length of stay”] OR [mh “patient readmission”] OR [mh “Clinical Laboratory Services”] OR [mh “Medication Reconciliation”] OR emergency care visit OR emergency department visit OR emergency department revisit OR emergency care revisit OR ED visit OR ED revisit OR medication OR medication utilisation OR pharmacy related practice OR pharmacy practice OR [mh “primary care”] OR primary care visit OR [mh “outpatient”] OR outpatient visit OR specialist clinic OR specialist care OR specialist care visit OR hospital admission OR [mh “hospital readmission”] OR hospital bed day OR bed day OR health care service OR [mh “health service”] OR [mh “day care”] OR [mh “ambulatory care”] OR [mh “Emergency department”] OR [mh “emergency care”] OR Hospitalization OR Hospitalisation OR length of stay OR patient readmission OR Medication Reconciliation OR primary care OR outpatient OR hospital readmission OR health service OR laboratory service OR day care OR ambulatory care Emergency department OR emergency care
7. 5 AND 6
8. systematic review OR meta-analysis OR meta-analyses
9. 7 AND 8
10. Limiters - Full Text; Publication Year: 2010-2022; Human; Language: English, Search modes - Boolean/Phrase

**OVID (MEDLINE R: EMBASE/APA Psychinfo/Cochrane database systematic review/EBM Reviews-Health Technology Assessment/EBM Reviews-NHS Economic Evaluation Database (NHSEED)**

1. Long-Term care/ or Insurance, Long-Term Care/ or long term care.mp. or long-term care.mp. or longterm care.mp. or aged care.mp.
2. Home Care Agencies/ or Home Health Aides/ or Home Care Services/ or social support/ or Assisted Living Facilities/ or Homes for the Aged/ or Nursing Homes/ or patient care.mp. or home care.mp. or formal care.mp. or home service*.mp. or social care.mp. or home.mp. and community based services.mp.) or community care.mp. or informal care.mp. or home care agencies.mp. or home health aides.mp. or home care service*.mp. or social support.mp. or assisted living facilities.mp. or homes for the aged.mp. or nursing home.mp.
3. 1 OR 2
4. Aged/ or Aged, 60.mp. and over/ or Frail Elderly/ or Geriatrics/ or elderly.mp. or elder person.mp. or old age.mp. or senior.mp. or older person.mp. or aged.mp. or old person.mp. or frail elderly.mp. or geriatric*.mp.
5. 3 AND 4
6. Hospitalization/ or Hospitalisation/ or length of stay/ or patient readmission/ or Clinical Laboratory Services/ or Medication Reconciliation/ or emergency care visit*.mp. or emergency department visit*.mp. or emergency department revisit*.mp. or emergency care revisit*.mp. or ED visit*.mp. or ED revisit*.mp. or medication.mp. or medication utilisation.mp. or pharmacy related practice.mp. or pharmacy practice.mp. or primary care/ or primary care visit.mp. or outpatient/ or outpatient visit*.mp. or specialist clinic.mp. or specialist care.mp. or specialist care visit*.mp. or hospital admission.mp. or hospital readmission/ or hospital bed day*.mp. or bed day*.mp. or health care service.mp. or health service/ or day care/ or ambulatory care/ or Emergency department/ or emergency care/ or Hospitalization.mp. or Hospitalisation.mp. or length of stay.mp. or patient readmission.mp. or Medication Reconciliation.mp. or primary care.mp. or outpatient*.mp. or hospital readmission.mp. or health service.mp. or laboratory service*.mp. or day care.mp. or ambulatory care.mp. or Emergency department.mp. or emergency care.mp.
7. 5 AND 6
8. systematic review.mp. or meta-analysis.mp. or meta-analyses.mp.
9. 8 AND 9
10. Limiters - Full Text; Publication Year: 2010-2022; Human; Language: English
