# Supplementary material for: The impact of long-term care interventions on healthcare utilisation among older persons: a scoping review of reviews
Source: BMC Geriatr. 2024 Jun 3;24:484. doi: 10.1186/s12877-024-05097-9 (PMC11145838; doi:10.1186/s12877-024-05097-9)
Supplement: Supplementary file 2 — Additional file 2. Template for extraction record [file 12877_2024_5097_MOESM2_ESM.docx]

**Additional file 2: Template for extraction record**

| **Bibliographic details** | | | | | | | | | | | | | | | |
| --- | --- | --- | --- | --- | --- | --- | --- | --- | --- | --- | --- | --- | --- | --- | --- |
| **Author** |  | | | | | | | | | | | | | | |
| **Year** |  | | | | | | | | | | | | | | |
| **Title** |  | | | | | | | | | | | | | | |
| **Journal** |  | | | | | | | | | | | | | | |
| **Review details** | | | | | | | | | | | | | | | |
| **Meta-analysis (Y/N)** | | | |  | | | | | | | | | | | |
| **Years of included studies** | | | |  | | | | | | | | | | | |
| **Study objective** | | | |  | | | | | | | | | | | |
| **Number of relevant studies and design** | | | |  | | | | | | | | | | | |
| **Number of participants** | | | |  | | | | | | | | | | | |
| **Population** | | | |  | | | | | | | | | | | |
| **Intervention** | | | |  | | | | | | | | | | | |
| **Comparison** | | | |  | | | | | | | | | | | |
| **Outcome**  (hospital admissions, hospital readmissions, ED visits, ED revisits, length of stay, primary care visits, drug use, outpatient visits, clinic visits, hospital bed days) | | | |  | | | | | | | | | | | |
| **Main findings** | | | | | | | | | | | | | | | |
| **For relevant studies:** | | | | | | | | | | | | | | | |
| **Author(s)** | **Study design** | | **Setting** | | **Sample size** | | | **Population** | | **Intervention** | | **Outcome** | | **AMSTAR-2 quality appraisal** | |
|  |  | |  | |  | | |  | |  | |  | |  | |
|  |  | |  | |  | | |  | |  | |  | |  | |
| **Summary table** | | | | | | | | | | | | | | | |
| **Intervention/Author** | | **Domain/Subdomain*** | | **Trials** | | **n** | **Follow-up** | | **Heterogeneity** | | **Effect size (95% CI)** | | **p-value** | | **GRADE (quality of evidence)** |
|  | |  | |  | |  |  | |  | |  | |  | |  |

*****Domain include (Compensate loss of capacity, prevent chronic condition, support capacity enhancing, manage chronic condition. promote capacity enhancing, early detection & control), Subdomain (Medication appropriateness, community-based complex intervention, Comprehensive Geriatric Assessment (CGA), transitional care, continuity of care, hospital care alternatives, home visit, community-based case management, coordinated/integrated care, perioperative geriatric management, community-based self-care, social care)
