# Supplementary figures and images for: The impact of long-term care interventions on healthcare utilisation among older persons: a scoping review of reviews
Source: BMC Geriatr. 2024 Jun 3;24:484. doi: 10.1186/s12877-024-05097-9 (PMC11145838; doi:10.1186/s12877-024-05097-9)

**Additional file 3: Flowchart of re-analysing extracted values**


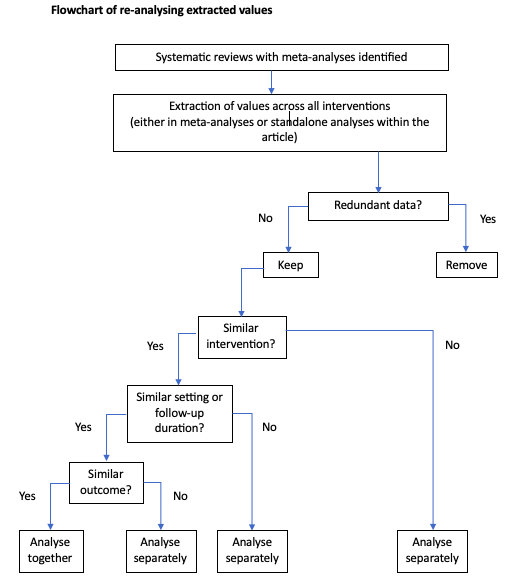

Supplement: Supplementary file 3 — Additional file 3. Flowchart for reanalysing extracted values [file 12877_2024_5097_MOESM3_ESM.docx]
