# Supplementary material for: The impact of long-term care interventions on healthcare utilisation among older persons: a scoping review of reviews
Source: BMC Geriatr. 2024 Jun 3;24:484. doi: 10.1186/s12877-024-05097-9 (PMC11145838; doi:10.1186/s12877-024-05097-9)
Supplement: Supplementary file 4 — Additional file 4. Excluded Articles [file 12877_2024_5097_MOESM4_ESM.docx]

**Additional file 4: Excluded Articles**

Articles are excluded for one of the following reasons:

1. Not older population
2. Not meta-analysis
3. Disease/condition specific
4. No related outcome

**Not older population (N = 6)**

1. Bryant-Lukosius D, Carter N, Reid K, Donald F, Martin-Misener R, Kilpatrick K, Harbman P, Kaasalainen S, Marshall D, Charbonneau-Smith R, DiCenso A. The clinical effectiveness and cost-effectiveness of clinical nurse specialist-led hospital to home transitional care: a systematic review. J Eval Clin Pract. 2015 Oct;21(5):763-81. doi: 10.1111/jep.12401.
2. English C, Shields N, Brusco NK, Taylor NF, Watts JJ, Peiris C, Bernhardt J, Crotty M, Esterman A, Segal L, Hillier S. Additional weekend therapy may reduce length of rehabilitation stay after stroke: a meta-analysis of individual patient data. J Physiother. 2016 Jul;62(3):124-9. doi: 10.1016/j.jphys.2016.05.015.
3. Smith TO, Sreekanta A, Walkeden S, Penhale B, Hanson S. Interventions for reducing hospital-associated deconditioning: A systematic review and meta-analysis. Arch Gerontol Geriatr. 2020 Sep-Oct;90:104176. doi: 10.1016/j.archger.2020.104176.
4. Caplan GA, Sulaiman NS, Mangin DA, Aimonino Ricauda N, Wilson AD, Barclay L. A meta-analysis of "hospital in the home". Med J Aust. 2012 Nov 5;197(9):512-9. doi: 10.5694/mja12.10480.
5. Taylor NF, Harding KE, Dennett AM, Febrey S, Warmoth K, Hall AJ, Prendergast LA, Goodwin VA. Behaviour change interventions to increase physical activity in hospitalised patients: a systematic review, meta-analysis and meta-regression. Age Ageing. 2022 Jan 6;51(1):afab154. doi: 10.1093/ageing/afab154.
6. Smith TO, Pearson M, Pfeiffer K, Crotty M, Lamb SE. Caregiver Interventions for Adults Discharged from the Hospital: Systematic Review and Meta-Analysis. J Am Geriatr Soc. 2019 Sep;67(9):1960-1969. doi: 10.1111/jgs.16048.

**Not meta-analysis (N = 7)**

1. Lambe K, Guerra S, Salazar de Pablo G, Ayis S, Cameron ID, Foster NE, Godfrey E, Gregson CL, Martin FC, Sackley C, Walsh N, Sheehan KJ. Effect of inpatient rehabilitation treatment ingredients on functioning, quality of life, length of stay, discharge destination, and mortality among older adults with unplanned admission: an overview review. BMC Geriatr. 2022 Jun 11;22(1):501. doi: 10.1186/s12877-022-03169-2.
2. Crocker TF, Clegg A, Riley RD, Lam N, Bajpai R, Jordão M, Patetsini E, Ramiz R, Ensor J, Forster A, Gladman JRF. Community-based complex interventions to sustain independence in older people, stratified by frailty: a protocol for a systematic review and network meta-analysis. BMJ Open. 2021 Feb 15;11(2):e045637. doi: 10.1136/bmjopen-2020-045637.
3. Veronese N, Custodero C, Demurtas J, Smith L, Barbagallo M, Maggi S, Cella A, Vanacore N, Aprile PL, Ferrucci L, Pilotto A; Special Interest Group in Systematic Reviews of the European Geriatric Medicine Society (EuGMS); Special Interest Group in Meta-analyses and Comprehensive Geriatric Assessment of the European Geriatric Medicine Society (EuGMS). Comprehensive geriatric assessment in older people: an umbrella review of health outcomes. Age Ageing. 2022 May 1;51(5):afac104. doi: 10.1093/ageing/afac104.
4. Tricco AC, Cogo E, Holroyd-Leduc J, Sibley KM, Feldman F, Kerr G, Majumdar SR, Jaglal S, Straus SE. Efficacy of falls prevention interventions: protocol for a systematic review and network meta-analysis. Syst Rev. 2013 Jun 6;2:38. doi: 10.1186/2046-4053-2-38.
5. Evans CJ, Ison L, Ellis-Smith C, Nicholson C, Costa A, Oluyase AO, Namisango E, Bone AE, Brighton LJ, Yi D, Combes S, Bajwah S, Gao W, Harding R, Ong P, Higginson IJ, Maddocks M. Service Delivery Models to Maximize Quality of Life for Older People at the End of Life: A Rapid Review. Milbank Q. 2019 Mar;97(1):113-175. doi: 10.1111/1468-0009.12373.
6. Cumal A, Colella TJF, Puts MT, Sehgal P, Robertson S, McGilton KS. The impact of facility-based transitional care programs on function and discharge destination for older adults with cognitive impairment: a systematic review. BMC Geriatr. 2022 Nov 14;22(1):854. doi: 10.1186/s12877-022-03537-y.
7. Dyer SM, Suen J, Williams H, Inacio MC, Harvey G, Roder D, Wesselingh S, Kellie A, Crotty M & Caughey GE. Impact of relational continuity of primary care in aged care: a systematic review. BMC Geriatr. 2022; 22: 579. doi: https://doi.org/10.1186/s12877-022-03131-2.

**Disease/condition specific (N = 6)**

1. Saripella A, Wasef S, Nagappa M, Riazi S, Englesakis M, Wong J, Chung F. Effects of comprehensive geriatric care models on postoperative outcomes in geriatric surgical patients: a systematic review and meta-analysis. BMC Anesthesiol. 2021 Apr 22;21(1):127. doi: 10.1186/s12871-021-01337-2.
2. Tam-Tham H, Cepoiu-Martin M, Ronksley PE, Maxwell CJ, Hemmelgarn BR. Dementia case management and risk of long-term care placement: a systematic review and meta-analysis. Int J Geriatr Psychiatry. 2013 Sep;28(9):889-902. doi: 10.1002/gps.3906.
3. Hshieh TT, Yang T, Gartaganis SL, Yue J, Inouye SK. Hospital Elder Life Program: Systematic Review and Meta-analysis of Effectiveness. Am J Geriatr Psychiatry. 2018 Oct;26(10):1015-1033. doi: 10.1016/j.jagp.2018.06.007.
4. Lee DA, Tirlea L, Haines TP. Non-pharmacological interventions to prevent hospital or nursing home admissions among community-dwelling older people with dementia: A systematic review and meta-analysis. Health Soc Care Community. 2020 Sep;28(5):1408-1429. doi: 10.1111/hsc.12984.
5. Pritchard E, Soh SE, Morello R, Berkovic D, Blair A, Anderson K, Bateman C, Moran C, Tsindos T, O'Donnell R, Ayton D. Volunteer Programs Supporting People With Dementia/Delirium in Hospital: Systematic Review and Meta-Analysis. Gerontologist. 2021 Nov 15;61(8):e421-e434. doi: 10.1093/geront/gnaa058
6. Lin S, Su S, Yeh W. Meta-Analysis: Effectiveness of Comprehensive Geriatric Care for Elderly Following Hip Fracture Surgery. West J Nurs Res. 2019;1-28. DOI: 10.1177/0193945919858715.

**No related outcome (N = 6)**

1. Berthelsen CB, Kristensson J. The content, dissemination and effects of case management interventions for informal caregivers of older adults: a systematic review. Int J Nurs Stud. 2015 May;52(5):988-1002. doi: 10.1016/j.ijnurstu.2015.01.006.
2. Martin RS, Hayes B, Gregorevic K, Lim WK. The Effects of Advance Care Planning Interventions on Nursing Home Residents: A Systematic Review. J Am Med Dir Assoc. 2016 Apr 1;17(4):284-93. doi: 10.1016/j.jamda.2015.12.017.
3. Frost R, Belk C, Jovicic A, Ricciardi F, Kharicha K, Gardner B, Iliffe S, Goodman C, Manthorpe J, Drennan VM & Walters K*.* Health promotion interventions for community-dwelling older people with mild or pre-frailty: a systematic review and meta-analysis. BMC Geriatr. 2017; 17:157. doi: https://doi.org/10.1186/s12877-017-0547-8.
4. Bachmann S, Finger C, Huss A, Egger M, Stuck AE, Clough-Gorr KM, et al. Inpatient rehabilitation specifically designed for geriatric patients: systematic review and meta-analysis of randomised controlled trials. BMJ. 2010; 340:c1718. doi:10.1136/bmj.c1718.
5. Carpenter CR, Shelton E, Fowler S, Suffoletto B, Platts-Mills TF, Rothman RE, Hogan TM. Risk factors and screening instruments to predict adverse outcomes for undifferentiated older emergency department patients: a systematic review and meta-analysis. Acad Emerg Med. 2015 Jan;22(1):1-21. doi: 10.1111/acem.12569.
6. Wallace E, Hinchey T, Dimitrov BD, Bennett K, Fahey T, Smith SM. A systematic review of the probability of repeated admission score in community-dwelling adults. J Am Geriatr Soc. 2013 Mar;61(3):357-64. doi: 10.1111/jgs.12150.
