# Supplementary material for: The impact of long-term care interventions on healthcare utilisation among older persons: a scoping review of reviews
Source: BMC Geriatr. 2024 Jun 3;24:484. doi: 10.1186/s12877-024-05097-9 (PMC11145838; doi:10.1186/s12877-024-05097-9)
Supplement: Supplementary file 5 — Additional file 5. AMSTAR-2 assessment of all included studies [file 12877_2024_5097_MOESM5_ESM.docx]

**Additional file 5: AMSTAR-2 assessment of all included studies**

**The AMSTAR-2 assessment was performed based on the recommendations following this reference:** Shea BJ, Reeves BC, Wells G, Thuku M, Hamel C, Moran J, et al. AMSTAR 2: a critical appraisal tool for systematic reviews that include randomised or non-randomised studies of healthcare interventions, or both. bmj. 2017;358. https://doi.org/10.1136/bmj.j4008

**According to the recommendation, items were marked as critical as follows:**

- Protocol registered before the commencement of the review (item 2)
- Adequacy of the literature search (item 4)
- Justification for excluding individual studies (item 7)
- Risk of bias from individual studies being included in the review (item 9)
- Appropriateness of meta-analytical methods (item 11)
- Consideration of risk of bias when interpreting the results of the review (item 13)
- Assessment of presence and likely impact of publication bias (item 15)

**The AMSTAR grade was assigned based on the following rating marks:**

•  High - No or one non-critical weakness: the systematic review provides an accurate and comprehensive summary of the results of the available studies that address the question of interest

•  Moderate - More than one non-critical weakness*: the systematic review has more than one weakness but no critical flaws. It may provide an accurate summary of the results of the available studies that were included in the review

•  Low - One critical flaw with or without non-critical weaknesses: the review has a critical flaw and may not provide an accurate and comprehensive summary of the available studies that address the question of interest

•  Critically low - More than one critical flaw with or without non-critical weaknesses: the review has more than one critical flaw and should not be relied on to provide an accurate and comprehensive summary of the available studies

*Multiple non-critical weaknesses may diminish confidence in the review, and moving the overall appraisal down from moderate to low confidence may be appropriate.

**Table S1: AMSTAR-2 assessment of all included studies**

| **Authors** | **Title** | **Year** | **Item** | | | | | | | | | | | | | | | | **Score (Critical items)** | **Score (Non-critical items)** | **AMSTAR grade** |
| --- | --- | --- | --- | --- | --- | --- | --- | --- | --- | --- | --- | --- | --- | --- | --- | --- | --- | --- | --- | --- | --- |
|  |  |  | **1** | **2** | **3** | **4** | **5** | **6** | **7** | **8** | **9** | **10** | **11** | **12** | **13** | **14** | **15** | **16** |  |  |  |
| Almutairi H, Stafford A, Etherton-Beer C, Flicker L. | Optimisation of medications used in residential aged care facilities: a systematic review and meta-analysis of randomised controlled trials | 2020 | Y | Y | Y | Y | N | Y | N | Y | Y | N | Y | Y | Y | Y | Y | Y | 1 | 2 | Low |
| Beswick AD, Gooberman-Hill R, Smith A, Wylde V, Ebrahim S | Maintaining independence in older people | 2010 | Y | N | Y | N | N | N | N | Y | N | N | Y | N | N | N | Y | N | 5 | 6 | Critical low |
| Briggs R, McDonough A, Ellis G, Bennett K, O'Neill D, Robinson D. | Comprehensive Geriatric Assessment for community-dwelling, highrisk, frail, older people | 2017 | Y | N | N | N | N | Y | Y | N | N | Y | Y | N | Y | N | N | Y | 4 | 5 | Critical low |
| Cheryl A. Sadowski* , Theresa L. Charrois, Evan Sehn, Trish Chatterley, Sujin Kim | The role and impact of the pharmacist in long-term care settings: A systematic review | 2020 | Y | Y | N | Y | Y | Y | N | N | N | N | N | Y | Y | N | N | Y | 4 | 4 | Critical low |
| Cochrane A, McGilloway S, Furlong M, Molloy W, Stevenson M, Donnelly M | Time-limited home-care reablement services for maintaining and improving the functional independence of older adults (Review) | 2016 | Y | Y | N | Y | Y | Y | Y | Y | Y | Y | Y | Y | Y | N | Y | N | 0 | 3 | Moderate |
| Conroy SP, Stevens T, Parker SG, Gladman JR. | A systematic review of comprehensive geriatric assessment to improve outcomes for frail older people being rapidly discharged from acute hospital: 'interface geriatrics' | 2011 | Y | N | N | N | N | Y | N | Y | N | N | N | N | N | N | Y | Y | 6 | 5 | Critical low |
| Deschodt MD, Buurman BM, Conroy SP, De Rooij SE | Impact of geriatric consultation teams on clinical outcome in acute hospitals: a systematic review and meta-analysis | 2014 | Y | Y | Y | Y | Y | Y | N | Y | N | N | Y | Y | Y | Y | Y | Y | 2 | 1 | Critical low |
| Deschodt M, Laurent G, Cornelissen L, Yip O, Zúñiga F, Denhaerynck K, Briel M, Karabegovic A, De Geest S | Core components and impact of nurse-led integrated care models for home-dwelling older people: A systematic review and meta-analysis | 2020 | Y | Y | Y | Y | Y | Y | N | Y | Y | N | Y | Y | Y | Y | N | Y | 2 | 1 | Critical low |
| Ekdahl AW, Sjöstrand F, Ehrenberg A, Oredsson S, Stavenow L, Wisten A, et al. | Frailty and comprehensive geriatric assessment organized as CGA-ward or CGA-consult for older adult patients in the acute care setting: A systematic review and meta-analysis | 2015 | Y | N | N | Y | Y | Y | N | Y | Y | N | Y | N | N | N | N | Y | 4 | 4 | Critical low |
| Ellis G, Gardner M, Tsiachristas A, Langhorne P, Burke O, Harwood RH, et al. | Comprehensive geriatric assessment for older adults admitted to hospital (Review) | 2017 | Y | N | N | Y | Y | Y | Y | Y | Y | N | Y | Y | Y | N | Y | Y | 1 | 3 | Low |
| Ellis G, Whitehead MA, O'Neill D, Langhorne P, Robinson D. | Comprehensive geriatric assessment for older adults admitted to hospital. | 2011 | Y | N | N | N | Y | Y | Y | Y | Y | Y | N | N | N | N | Y | Y | 4 | 3 | Critical low |
| Ellis G, Whitehead MA, Robinson D, O'Neill D, Langhorne P | Comprehensive geriatric assessment for older adults admitted to hospital: Meta-analysis of randomised controlled trials | 2011 | Y | N | Y | Y | Y | Y | N | Y | Y | N | Y | Y | Y | Y | N | Y | 3 | 1 | Critical low |
| Facchinetti G, D'Angelo D, Piredda M, Petitti T, Matarese M, Oliveti A, et al | Continuity of care interventions for preventing hospital readmission of older people with chronic diseases: A meta-analysis | 2020 | Y | N | N | N | Y | Y | N | Y | N | N | N | N | Y | Y | Y | Y | 5 | 3 | Critical low |
| Forster A, Young J, Lambley R, Langhorne P | Medical day hospital care for the elderly versus alternative forms of care (Review) | 2010 | Y | N | N | Y | Y | Y | Y | Y | Y | N | Y | N | N | N | N | Y | 3 | 4 | Critical low |
| Fox MT, Persaud M, Maimets I, O'Brien K, Brooks D, Tregunno D, et al. | Effectiveness of acute geriatric unit care using Acute Care for Elders components: A systematic review and meta-analysis | 2012 | Y | N | Y | Y | Y | Y | N | Y | Y | N | Y | Y | Y | Y | N | Y | 3 | 1 | Critical low |
| Hill-Taylor B, Walsh KA, Stewart S, Hayden J, Byrne S, Sketris S. | Effectiveness of the STOPP/START (Screening tool of older persons' potentially inappropriate prescriptions/ screening tool to alert doctors to the right treatment) criteria: Systematic review and meta-analysis of randomized controlled studies | 2016 | Y | Y | Y | Y | Y | Y | N | Y | Y | Y | Y | Y | N | N | N | Y | 3 | 1 | Critical low |
| Lee SWH, Mak VSL, Tang YW | Pharmacist services in nursing homes: A systematic review and meta-analysis | 2019 | Y | N | Y | Y | Y | Y | N | Y | Y | N | Y | Y | Y | Y | Y | Y | 2 | 1 | Critical low |
| Lin SN, Su SF, Yeh WT. | Meta-analysis: Effectiveness of Comprehensive Geriatric Care for Elderly Following Hip Fracture Surgery. | 2020 | Y | N | Y | Y | N | Y | N | N | N | N | Y | Y | Y | N | Y | Y | 3 | 4 | Critical low |
| Lowthian JA, McGinnes RA, Brand CA, Barker AL, Brand CA, Cameron PA | Effective discharge of older patients from the emergency department: Systematic review and meta-analysis | 2015 | Y | Y | Y | Y | Y | Y | N | Y | Y | N | Y | Y | Y | Y | N | Y | 2 | 1 | Critical low |
| Luker JA, Worley A, Stanley M, Uy J, Watt AM, Hillier SL. | The evidence for services to avoid or delay residential aged care admission: a systematic review | 2019 | Y | Y | Y | Y | Y | N | N | Y | Y | N | Y | Y | Y | Y | N | Y | 2 | 2 | Critical low |
| Mayo-Wilson E, Grant S, Burton J, Montgomery P, Parsons A, Underhill K | Preventive Home Visits for Mortality, Morbidity, and Institutionalization in Older Adults: A Systematic Review and Meta-Analysis | 2014 | Y | N | N | N | N | N | Y | Y | Y | N | Y | N | N | Y | N | Y | 4 | 5 | Critical low |
| Poupard N, Tang CY, Shields N | Community-based case management does not reduce hospital admissions for older people: a systematic review and meta-analysis | 2020 | Y | Y | Y | Y | N | Y | Y | Y | Y | N | Y | Y | Y | Y | N | Y | 2 | 1 | Critical low |
| Rodakowski J, Rocco PB, Ortiz M, Folb B, Schulz R, Morton SC, et al | Caregiver integration during discharge planning for older adults to reduce resource use: A metaanalysis | 2017 | Y | Y | Y | Y | Y | Y | N | Y | Y | N | Y | Y | Y | Y | Y | Y | 1 | 1 | Critical low |
| Spiers G, Matthews FE, Barker RO, Jarvis H, Stow D, Kingston A, et al | Impact of social care supply on healthcare utilisation by older adults: A systematic review and meta-analysis | 2019 | Y | N | N | Y | N | N | Y | N | N | N | N | N | N | N | N | Y | 5 | 7 | Critical low |
| Tecklenborg S, Cahir C, Brown L, Bennett K, Byrne C | Interventions to Reduce Adverse Drug Event‑Related Outcomes in Older Adults: A Systematic Review and Meta‑analysis | 2020 | Y | N | N | Y | Y | N | N | Y | N | N | N | N | N | N | N | Y | 6 | 5 | Critical low |
| Thillainadesan J, Yumol MF, Hilmer S, Aitken SJ, Naganathan V. | Interventions to Improve Clinical Outcomes in Older Adults Admitted to a Surgical Service: A Systematic Review and Meta-analysis | 2020 | Y | N | N | Y | Y | Y | N | Y | N | N | Y | Y | N | N | Y | Y | 4 | 3 | Critical low |
| Wallerstedt SM, Kindblom JM, Nylen K, Samuelsson O, Strandell A | Medication reviews for nursing home residents to reduce mortality and hospitalization: Systematic review and meta-analysis | 2014 | Y | N | N | Y | Y | N | N | Y | N | N | N | N | N | N | N | Y | 6 | 5 | Critical low |
| Weeks LE, Macdonald M, Martin-Misener R, Bishop A, Iduye DF, Helwig M, et al | The impact of transitional care programs on health services utilization in community-dwelling older adults: A systematic review | 2018 | Y | N | N | Y | N | N | Y | Y | N | N | N | N | N | N | N | Y | 5 | 6 | Critical low |
| Wong KC, Wong FKY, Yeung WF, Chang K | The effect of complex interventions on supporting self-care among community-dwelling older adults: A systematic review and meta-analysis | 2018 | Y | Y | Y | Y | Y | Y | N | Y | Y | N | Y | Y | Y | Y | Y | Y | 1 | 1 | Low |
