# Supplementary material for: The impact of long-term care interventions on healthcare utilisation among older persons: a scoping review of reviews
Source: BMC Geriatr. 2024 Jun 3;24:484. doi: 10.1186/s12877-024-05097-9 (PMC11145838; doi:10.1186/s12877-024-05097-9)
Supplement: Supplementary file 6 — Additional file 6. Summary of associations between long-term care interventions with hospital utilisation among older persons [file 12877_2024_5097_MOESM6_ESM.docx]

**Additional file 6: Summary of associations between long-term care interventions with hospital utilisation among older persons**

| **Interventions** | **Domain/ Subdomain** | **Study design** | **Trials** | **n** | **Follow-up (months)** | **Heterogeneity (I^2^), %** | **Effect size, Random (95% CI)** | **p-value** | **GRADE rating** |
| --- | --- | --- | --- | --- | --- | --- | --- | --- | --- |
| **Hospital Admission** | | | | | | | | |  |
| Community-based case management  Poupard et al. (2019) | Manage chronic conditions;  Community-based case management | RCT | 2 | 200 | 12 | 62.3 | MD: -0.03 (-0.28, 0.23) | 0.839 | Low |
|  |  | OBS | 1 | 369 | 12 | - | OR: 0.65 (0.41, 1.02) | 0.059 | Very low |
|  |  | RCT | 1 | 139 | 14 | - | MD: 0.10 (-0.15, 0.35) | 0.433 | Low |
| Medication review by pharmacist in LTC  Sadowski et al. (2020) | Manage chronic conditions; Medication appropriateness | RCT | 1 | 661 | 6 | - | OR: 0.89 (0.58, 1.36) | 0.575 | Very low |
|  |  | RCT | 2 | 169 | 12 | 10.4 | **OR: 0.16 (0.03, 0.73)** | **0.019** | Very low |
|  |  | OBS | 2 | 4054 | 12 | 67.2 | OR: 0.84 (0.60, 1.16) | 0.284 | Very low |
| Medication reviews in nursing homes  Wallerstedt et al. (2014) | Manage chronic conditions; Medication appropriateness | RCT | 2 | 886 | 6 | 35.6 | OR: 1.07 (0.58, 2.01) | 0.822 | Very low |
| Interventions to reduce ADEs in primary care  Tecklenborg et al. (2020) | Manage chronic conditions; Medication appropriateness | MIX | 2 | 570 | 3 | 0 | RR: 0.87 (0.52, 1.47) * | Not sig. | Very low |
|  |  | RCT | 2 | 586 | 12 | 95.3 | SMD: -0.41 (-1.22, 0.40) * | Not sig. | Very low |
| Pharmacists' services in nursing home  Lee et al. (2019) | Manage chronic conditions; Medication appropriateness | RCT | 2 | 749 | 1-6 | 66.1 | OR: 0.60 (0.22, 1.63) | 0.313 | Very low |
|  |  | OBS | 1 | 135 | 12 | - | OR: 0.29 (0.06, 1.42) | 0.125 | Very low |
| Medication optimisation in residential aged/continuing care  Almutairi et al. (2020) | Manage chronic conditions; Medication appropriateness | RCT | 4 | 1690 | 1-6 | 48.7 | OR: 0.88 (0.55, 1.42) | 0.608 | Very low |
|  |  | RCT | 3 | 3720 | 12 | 0 | OR: 1.05 (0.88, 1.27) | 0.584 | Low |
|  |  | RCT | 1 | 1998 | 14 | - | OR: 0.92 (0.77, 1.10) | 0.379 | Moderate |
| Deprescribing Interventions  Kua et al. (2019) | Manage chronic conditions; Medication appropriateness | RCT | 3 | 907 | 1-6 | 65.5 | OR: 0.89 (0.30, 2.69) | 0.842 | Very low |
|  |  | RCT | 1 | 95 | 12 | - | **OR: 0.40 (0.17, 0.92)** | **0.031** | **Very low** |
| Anti-microbial stewardship  Crespo-Rivas et al. (2021) | Manage chronic conditions; Medication appropriateness | RCT | 3 | 84 | 12 | 17 | MD: 0.17 (-0.07, 0.41)* | 0.18 | Very low |
| Geriatric patient care by pharmacist  Lee et al. (2013) | Manage chronic conditions; Medication appropriateness | OBS | 2 | 879 | 12-24 | - | NR | **2 trials showed significant reductions**  **(0.03 & 0.00)** | **Very low** |
| ED community transition strategies  Lowthian et al. (2015) | Support capacity enhancing; Transitional care | RCT | 2 | 1389 | 1 | 0 | OR: 0.89 (0.65, 1.21) | 0.459 | Low |
|  |  | OBS | 1 | 1724 |  | 0 | OR: 0.91 (0.59, 1.41) | 0.681 | Very low |
| Nurse-led integrated care models  Deschodt et al. (2020) | Support capacity enhancing; Coordinated/Integrated care | MIX | 11 | 16,942 | 3-24 | 4.7 | OR: 0.94 (0.87–1.02) * | Not sig. | Very low |
| Community-based complex interventions  Wong et al. (2018) | Promote capacity enhancing; Community-based self-care | RCT | 3 | 1392 | 7-12 | 53.6 | OR: 0.82 (0.55, 1.22) | 0.328 | Very low |
|  |  | RCT | 5 | 3105 | 13-24 | 29.4 | OR: 1.06 (0.87, 1.27) | 0.573 | Very low |
| Community-based multifactorial interventions  Beswick et al. (2010) | Promote capacity enhancing; Community-based complex intervention | RCT | 51 | 24,424 | 1-6 | 44.3 | RR: 0.94 (0.92, 0.97) * | NR | Very low |
| Community-based, aged-care interventions  Luker et al. (2019) | Promote capacity enhancing; Community-based complex intervention | RCT | 1 | 739 | 18 | - | **OR: 0.67 (0.50, 0.89)** | **0.006** | **Very low** |
|  |  | RCT | 1 | 294 | 24 | - | **MD: -0.38 (-0.69, -0.07)** | **0.016** | **Very low** |
| Preventive home visit  Mayo-Wilson et al. (2014) | Promote capacity enhancing; home visit | RCT | 2 | 332 | 1-6 | 0 | OR: 0.56 (0.27, 1.14) | 0.110 | Low |
|  |  | RCT | 7 | 2155 | 7-12 | 0 | **OR: 0.73 (0.59, 0.91)** | **0.005** | **Moderate** |
|  |  | RCT | 5 | 1937 | 13-24 | 47.4 | OR: 1.04 (0.75, 1.45) | 0.823 | Very low |
|  |  | RCT | 4 | 3438 | 36 | 0 | OR: 0.88 (0.77, 1.01) | 0.063 | High |
| CGA in a community setting  Briggs et al. (2022) | Early Detection and Control; CGA | RCT | 3 | 901 | 7-12 | 71.4 | OR: 0.76 (0.45, 1.31) | 0.330 | Very low |
|  |  | RCT | 2 | 583 | 13-24 | 0 | **OR: 0.57 (0.41, 0.80)** | **0.001** | **Low** |
|  |  | RCT | 1 | 232 | 36 | - | OR: 0.92 (0.54, 1.57) | 0.764 | Low |
| Time-limited home-care reablement services  Cochrane et al. (2016) | Compensate Loss of Capacity | RCT | 1 | 750 | 12 | - | OR: 0.88 (0.66, 1.17) | 0.377 | Very low |
|  |  | RCT | 1 | 750 | 24 | - | OR: 0.81 (0.60, 1.10) | 0.182 | Very low |
| **Hospital Readmission** | | | | | | | | |  |
| Caregiver integration during discharge planning  Rodakowski et al. (2017) | Support capacity enhancing; Coordinated/Integrated care | RCT | 13 | 4374 | 1-6 | 32.0 | **OR: 0.68 (0.57, 0.81)** | **0.000** | **High** |
|  |  | RCT | 2 | 481 | 1-6 | 85.9 | MD: -0.05 (-0.27, 0.16) | 0.631 | Very low |
|  |  | RCT | 3 | 1325 | 7-12 | 39.1 | OR: 0.67 (0.43, 1.05) | 0.080 | Moderate |
| In-hospital geriatric co-management  Van Grootven et al. (2017) | Support capacity enhancing; Coordinated/Integrated care | OBS | 3 | 685 | 1-6 | 24.2 | OR: 1.24 (0.60, 2.57) | 0.568 | Very low |
|  |  | RCT | 1 | 108 | 7-12 | 0.0 | OR: 1.11 (0.52, 2.38) | 0.795 | Very low |
|  |  | OBS | 1 | 464 | 7-12 | 0.0 | OR: 0.86 (0.58, 1.27) | 0.453 | Very low |
| Early supported discharge  William et al. (2022) | Support capacity enhancing; Transitional care | RCT | 2 | 389 | 1-6 | 29.3 | OR: 1.20 (0.65, 2.21) | 0.570 | Very low |
|  |  | RCT | 1 | 279 | 12 | - | OR: 0.99 (0.60, 1.65) | 0.968 | Very low |
| Transitional care programs for community-dwelling older adults  Weeks et al. (2018) | Support capacity enhancing; Transitional care | RCT | 10 | 7751 | 1-6 | 77.4 | **OR: 0.79 (0.62, 1.00)** | **0.048** | **Very low** |
|  |  | OBS | 2 | 2537 | 1-6 | 22.6 | **OR: 0.54 (0.38, 0.76)** | **0.000** | **Very low** |
| Transitional care programs for LTCF residents  Birtwell et al. (2022) | Support capacity enhancing; Transitional care | MIX | 11 | NR | 1-6 | 40.0 | **OR: 1.48 (1.01, 2.17) *** | **Sig. (likelihood not to be readmitted)** | **Very low** |
| Integrating primary healthcare in aftercare  Ran Li et al. (2022) | Support capacity enhancing; Continuity of care | RCT | 22 | 3990 | 1-6 | 71.4 | **OR: 0.60 (0.49, 0.74)** | **0.000** | **Low** |
| Enhanced medication continuity  Tomlinson et al. (2020) | Support capacity enhancing; Continuity of care | RCT | 13 | 9070 | 1-6 | 76 | OR: 0.87 (0.69, 1.10) | 0.252 | Very low |
|  |  | RCT | 3 | 1285 | 12 | 58.9 | OR: 0.73 (0.49, 1.07) | 0.108 | Very low |
| Continuity of care  Facchinetti et al. (2020) | Support capacity enhancing; Continuity of care | RCT | 21 | 6407 | 1-6 | 64.0 | **OR: 0.80 (0.66, 0.97)** | **0.026** | **Very low** |
|  |  | RCT | 12 | 2066 | 7-12 | 30.8 | **OR: 0.76 (0.61, 0.95)** | **0.018** | **Moderate** |
| Home-based exercise programmes  Ingrid Lin et al. (2022) | Promote capacity enhancing | RCT | 4 | 472 | 1-6 | 58.7 | OR: 0.73 (0.38, 1.40) | 0.340 | Very low |
| CGA-post hospital discharge model  Conroy et al. (2011) | Early detection & control; CGA | RCT | 3 | 1664 | 1-6 | 78.4 | OR: 0.68 (0.40, 1.15) | 0.147 | Low |
|  |  | RCT | 2 | 710 | 7-12 | 0.0 | OR: 0.73 (0.51, 1.04) | 0.082 | Low |
| CGA-ward  O' Shaughnessy et al. (2022) | Early detection & control; CGA | RCT | 7 | 6236 | 1-6 | 13.3 | OR: 1.03 (0.89, 1.19) | 0.704 | Very low |
| CGA-ward  Fox et al. (2012) | Early detection & control; CGA | RCT | 5 | 3983 | 1-6 | 0.0 | OR: 1.06 (0.90, 1.25) | 0.484 | Low |
| CGA-consult  Deschodt et al. (2013) | Early detection & control; CGA | RCT | 3 | NR | 6 | 86 | RR: 0.88 (0.40, 1.96)* | 0.76 | Very low |
|  |  | MIX | 5 | NR | 12 | 12 | RR: 0.99 (0.83, 1.19)* | 0.94 | Low |
| CGA-ward and CGA-consult  Ellis et al. (2011) | Early detection & control; CGA | RCT | 9 | 3822 | - | - | OR: 1.03 (0.89, 1.18)* | 0.72 | Low |
| Perioperative geriatric interventions  Thillainadesan et al. (2020) | Prevent chronic condition; Perioperative geriatric management | RCT | 3 | 519 | 1-6 | 0.0 | OR: 1.58 (0.98, 2.56) | 0.060 | Very low |
|  |  | OBS | 2 | 311 | 1-6 | 80.1 | OR: 0.58 (0.09, 3.72) | 0.563 | Very low |
| Geriatric patient care by pharmacist  Lee et al. (2013) | Manage chronic condition; Medication appropriateness | OBS | 2 | 943 | 1-2 | - | NR | **1/2 trials showed significant reduction in intervention group**  **(0.040)** | Very low |
| Community-based, aged-care interventions  Luker et al. (2019) | Promote capacity enhancing; Community-based complex intervention | RCT | 1 | 412 | 6 | - | **RR: 1.30 (1.07, 1.58)*** | **0.009** | **Low** |
|  |  | RCT | 1 | NR | 12 | - | NR | **Intervention group had fewer readmissions (0.03)** | **Low** |
| Social care  Spiers et al. (2019) | Promote Capacity Enhancing; Social Care | OBS | 3 | NR | NR | - | NR | Intervention produced fewer emergency readmission | Very low |
| CGA-Ward  Ekhdal et al. (2015)  Ellis et al. (2017) | Early detection & control; CGA | RCT | 11 | 5992 | 1-12 | 0.0 | OR: 1.02 (0.90, 1.15) | 0.818 | High |
| CGA-Consult  Ekhdal et al. (2015)  Ellis et al. (2017) | Early detection & control; CGA | RCT | 6 | 2980 | 1-12 | 47.3 | OR: 0.93 (0.72, 1.21) | 0.602 | Very low |
| **Length of stay (LOS)** | | | | | | | | |  |
| Community-based case management  Poupard et al. (2019) | Manage chronic condition;  Community-based case management | RCT | 2 | 292 | 12-14 | 65 | MD: 3.69 (-3.77, 11.15) | 0.332 | Very low |
| Geriatric patient care by pharmacist  Lee et al. (2013) | Manage chronic condition; Medication appropriateness | OBS | 2 | 584 | Hosp | - | NR | ½ trial shows significant reduction | Very low |
| Caregiver integration during discharge planning  Rodakowski et al. (2017) | Support capacity enhancing; Coordinated/Integrated care | RCT | 7 | NR | 1-12 |  | NR | 6/7 trials showed reduction, 5 are significant | Low |
| In-hospital geriatric co-management  Van Grootven et al. (2017) | Support capacity enhancing; Coordinated/Integrated care | RCT | 6 | 1236 | Hosp | 89.1 | MD: -5.64 (-12.47, 1.19) | 0.106 | **Very low** |
|  |  | OBS | 5 | 2158 |  | 82.9 | MD: -1.73 (-3.81, 0.35) | 0.103 | **Very low** |
| Transitional care programs for LTCF residents  Birtwell et al. (2022) | Support capacity enhancing; Transitional care | MIX | 7 | NR | NR | 98 | SMD: -1.86 (-5.47, 1.75)* | Not sig. | Very low |
| Transitional care programs for community-dwelling older adults  Weeks et al. (2018) | Support capacity enhancing; Transitional care | RCT | 7 | NR | 1-36 |  | NR | 6/7 trials showed reduction, 3 are significant | Low |
| Early supported discharge  William et al. (2022) | Support capacity enhancing; Transitional care | RCT | 4 | 1023 | Hosp | 90.1 | **MD: -6.04 (-9.76, -2.32)** | **0.001** | **Very low** |
| Integrating primary healthcare in aftercare  Ran Li et al. (2022) | Support capacity enhancing; Continuity of care | RCT | 14 | 3283 | Hosp | 52.6 | MD: 0.03 (-0.63, 0.68) | 0.940 | Very low |
| Perioperative geriatric interventions  Thillainadesan et al. (2020) | Prevent Chronic Condition; Perioperative geriatric management | RCT | 8 | 1179 | pre/post operative | 0 | **MD: -1.57 (-2.21, -0.93)** | **0.000** | **Moderate** |
|  |  | OBS | 4 | 453 |  | 31.8 | MD: -1.51 (-3.62, 0.60) | 0.161 | Very low |
| CGA-consult  Deschodt et al. (2013) | Early Detection and Control; CGA | MIX | 9 | 1782 | Hosp, 1-12 | 0 | MD: -0.35 (-1.24 to 0.55) * | 0.450 | Very low |
| CGA-ward and  CGA-consult  Ellis et al. (2017) | Early Detection and Control; CGA | RCT | 17 | 5303 | Hosp, 3-12 | 80 | MD: 0.69 (-0.580 1.95) | 0.288 | Very low |
| CGA-ward  Fox et al. (2012) | Early detection & control; CGA | RCT | 7 | 5128 | Hosp, 3 | 51.7 | **MD: -0.62 (-1.24, -0.01)** | **0.047** | **Very low** |
|  |  | OBS | 4 | 970 |  | 95.1 | MD: -2.26 (-6.42, 1.89) | 0.286 | Very low |
| CGA-ward  O' Shaughnessy et al. (2022) | Early detection & control; CGA | RCT | 9 | 6646 | Hosp | 76.8 | MD: -0.36 (-0.99, 0.26) | 0.256 | Very low |
| Social care  Spiers et al. (2019) | Promote Capacity Enhancing; Social Care | OBS | 4 | NR | NR | - | NR | Intervention reduce LOS | Very low |
| **Bed days** | | | | | | | | |  |
| Community-based case management  Poupard et al. (2019) | Manage chronic condition;  Community-based case management | RCT | 2 | 445 | 12-36 | 51.2 | MD: -4.02 (-8.38, 0.33) | 0.070 | Very low |
| Medical day hospital  Forster et al. (2008) | Manage chronic conditions; Hospital care alternatives | RCT | 13 | NR | 1-12 |  | NR | 7/13 trials showed reduction in bed days/pt | Very low |

*Value taken from meta paper due to insufficient data for reanalysis

LTC: long-term care, LTCF: long-term care facility, ADE: adverse drug event, CGA: comprehensive geriatric assessment, Hosp: hospitalisation, MD: mean difference, SMD: standardised mean difference, OR: odd ratio, RR: risk ratio, 95% CI: 95% confidence Interval, n = total number of participants in trials, pt: patients, sig.: significant, NR: not reported, RCT: Randomised controlled trial, OBS: Observational study, MIX: RCT and OBS

GRADE: Grading of Recommendations, Assessment, Development and Evaluations

**Supplementary Table S2.** Summary of associations between long-term care interventions with emergency department utilisation among older persons

| **Study** | **Domain/ Subdomain** | **Study design** | **Trials** | **n** | **Follow-up (months)** | **Heterogeneity (I^2^), %** | **Effect size, Random, (95% CI)** | **p-value** | **GRADE rating** |
| --- | --- | --- | --- | --- | --- | --- | --- | --- | --- |
| **Emergency Department Visits** | | | | | | | | |  |
| Community-based case management  Poupard et al. (2019) | Manage chronic condition; Community-based case management | RCT | 1 | 139 | 6 | - | MD: -0.38 (-0.86, 0.10) | 0.118 | Very low |
|  |  | RCT | 1 | 92 | 12 | - | **MD: -0.50 (-0.96, -0.04)** | **0.034** | **Very low** |
|  |  | RCT | 1 | 108 | 14 | - | MD: -0.10 (-0.38, 0.18) | 0.475 | Low |
| STOPP/START  Hill-Taylor et al. (2016) | Manage chronic condition; Medication appropriateness | RCT | 1 | NR | NR | - | NR | Increased visits in control group while no changes in intervention group **(0.022)** | **Very low** |
| Interventions to reduce incidence of ADEs  Tecklenborg et al. (2020) | Manage chronic condition; Medication appropriateness | RCT | 2 | NR | 1-6 | 0.0 | SMD: 0.06 (-0.07, 0.20)* | 0.972 | Low |
| CGA in community setting  Briggs et al. (2022) | Early detection & control; CGA | RCT | 1 | 199 | 12 | - | **OR: 0.32 (0.12, 0.84)** | **0.020** | **Low** |
|  |  | RCT | 1 | 423 | 24 | - | OR: 1.43 (0.82, 2.49) | 0.213 | Very low |
| Nurse-led integrated care models  Deschodt et al. (2020) | Support capacity enhancing; Coordinated/Integrated care | MIX | 7 | 16,834 | 1-24 | 61.7 | OR: 1.00 (0.84, 1.19)* | Not sig. | Very low |
| Home-care reablement services  Cochrane et al. (2016) | Compensate loss of capacity | RCT | 1 | 750 | 12 | - | OR: 0.81 (0.61, 1.08) | 0.144 | Very low |
|  |  | RCT | 1 | 750 | 24 | - | OR: 0.81 (0.60, 1.09) | 0.165 | Very low |
| Home-visit  Mayo-Wilson et al. (2014) | Promote capacity enhancing; Home visit | RCT | 9 | 3080 | 1-12 | 12.5 | OR: 1.00 (0.80, 1.18) | 0.734 | Very low |
|  |  | RCT | 3 | 1720 | 13-24 | 83.6 | OR: 1.05 (0.64, 1.73) | 0.848 | Very low |
| Transitional care programs for community-dwelling older adults  Weeks et al. (2018) | Support capacity enhancing; Transitional care | RCT | 5 | NR | 1-12 |  | NR | 1/5 trials showed significant reduction of outcome at 3 months **(0.03)** | **Low** |
| **Length of Stay (LOS)** | | | | | | | | |  |
| Transitional care programs for LTCF residents  Birtwell et al. (2022) | Support capacity enhancing; Transitional care | MIX | 3 | 679 | NR | 99 | **SMD: -3.51 (-3.61, -2.39)*** | **Sig.** | **Very low** |
| **Emergency Department Revisits** | | | | | | | | |  |
| ED-community transition strategies  Lowthian et al. (2015) | Support capacity enhancing; Transitional care | RCT | 2 | 1389 | 1 | 0.0 | OR: 1.32 (0.99, 1.76) | 0.062 | Low |
|  |  | OBS | 2 | 3920 | 1 | 86.1 | OR: 0.99 (0.62, 1.59) | 0.969 | Very low |
| Transitional care programs for LTCF residents  Birtwell et al. (2022) | Support capacity enhancing; Transitional care | MIX | 5 | NR | NR | 93.0 | OR: 2.04 (0.96, 4.33)* | Not sig. | Very low |

*Value taken from meta paper due to insufficient data for reanalysis

ED: emergency department, LTCF: long-term care facility, ADE: adverse drug event, STOPP/START: Screening Tool of Older Person's potentially inappropriate Prescriptions/Screening Tool to Alert doctors to the Right Treatment, CGA: comprehensive geriatric assessment, MD: mean difference, SMD: standardised mean difference, OR: odd ratio, RR: risk ratio, 95% CI: 95% confidence interval, n = total number of participants in trials, sig.: significant, NR: not reported

RCT: Randomised controlled trial, OBS: Observational study, MIX: RCT and OBS

GRADE: Grading of Recommendations, Assessment, Development and Evaluations

**Supplementary Table S3.** Summary of associations between long-term care interventions with medication utilisation among older persons

| **Study** | **Domain/ Subdomain** | **Study design** | **Trials** | **n** | **Follow-up (months)** | **Heterogeneity (I^2^), %** | **Effect size, Random, (95% CI)** | **p-value** | **GRADE rating** |
| --- | --- | --- | --- | --- | --- | --- | --- | --- | --- |
| **Drug Use** | | | | | | | | |  |
| STOPP/START  Hill-Taylor et al. (2016) | Manage chronic conditions; Medication appropriateness | RCT | 2 | NR | NR | - | NR | 1/2 trials had greater reduction in mean number of drugs for intervention group | Very low |
|  |  | RCT | 1 | NR | NR | - | NR | Polypharmacy of >6 and > 11 drugs reduced in intervention group | Low |
|  |  | RCT | 1 | NR | NR | - | NR | Antipsychotic use declined for intervention group | Low |
|  |  | RCT | 1 | 385 | NR | - | NR | Both the intervention and control groups had increased duplicate medications | Low |
| Anti-microbial stewardship  Crespo-Rivas et al. (2021) | Manage Chronic Conditions; Medication appropriateness | RCT | 3 | 84 | 12 | 71 | **MD: -0.47 (-0.87, -0.07)*** | **0.02** | **Very low** |
| Geriatric patient care by pharmacist  Lee et al. (2013) | Manage chronic conditions; Medication appropriateness | OBS | 4 | 4643 | NR | - | NR | 2/4 trials had significant reduction | Very low |

*Value taken from meta paper due to insufficient data for reanalysis

STOPP/START: Screening Tool of Older Person's potentially inappropriate Prescriptions/Screening Tool to Alert doctors to the Right Treatment, MD: mean difference, 95% CI: 95% confidence interval, n = total number of participants in trials, sig.: significant, NR: not reported

RCT: Randomised controlled trial, OBS: Observational study

GRADE: Grading of Recommendations, Assessment, Development and Evaluations

**Supplementary Table S4.** Summary of associations between long-term care interventions with primary care utilisation among older persons

| **Study** | **Domain/ Subdomain** | **Study design** | **Trials** | **n** | **Follow-up (months)** | **Heterogeneity (I^2^), %** | **Effect size, Random, (95% CI)** | **p-value** | **GRADE rating** |
| --- | --- | --- | --- | --- | --- | --- | --- | --- | --- |
| **Primary care visit** | | | | | | | | |  |
| Community-based, aged care interventions  Luker et al. (2019) | Promote capacity enhancing; Community-based complex intervention | RCT | 1 | NR | NR | - | **RR: 1.43 (1.14 to 1.80)*** | **0.002** | **Very low** |
| STOPP/START  Hill-Taylor et al. (2016) | Manage chronic condition; Medication appropriateness | RCT | 2 | NR | NR | - | NR | 2 trials show primary care visit reduction in intervention group (1 sig.) | Low |
| Transitional care programs for community-dwelling older adults  Weeks et al. (2018) | Support Capacity Enhancing; Transitional Care | MIX | 8 | NR | NR | - | NR | 2 trials showed significantly higher primary care visit in intervention group | Very low |

*Value taken from meta paper due to insufficient data for reanalysis

RCT: randomised control trial, OBS: observational study, MIX: mixed study design

STOPP/START: Screening Tool of Older Person's potentially inappropriate Prescriptions/Screening Tool to Alert doctors to the Right Treatment, RR: risk ratio, 95% CI: 95% confidence interval, n = total number of participants in trials, sig.: significant, NR: not reported

RCT: Randomised controlled trial, OBS: Observational study, MIX: RCT and OBS

GRADE: Grading of Recommendations, Assessment, Development and Evaluations
