# Supplementary material for: The impact of long-term care interventions on healthcare utilisation among older persons: a scoping review of reviews
Source: BMC Geriatr. 2024 Jun 3;24:484. doi: 10.1186/s12877-024-05097-9 (PMC11145838; doi:10.1186/s12877-024-05097-9)
Supplement: Supplementary file 7 — Additional file 7. GRADE assessment for significant associations [file 12877_2024_5097_MOESM7_ESM.docx]

**Additional file 7: GRADE assessment for significant associations**

| **Intervention** | **No of trials** | **Follow-up** | **Study design** | **Risk of bias** | **Inconsistency** | **Indirectness** | **Imprecision** | **Publication bias** | **Certainty** |
| --- | --- | --- | --- | --- | --- | --- | --- | --- | --- |
| **Hospital utilisation** | | | | | | | | | |
| **Hospital admission** | | | | | | | | | |
| Deprescribing Interventions Kua et al. (2019) | 1 | 12 | RCT | Serious^a^ | not serious | not serious | very serious ^e,g^ | undetected | Very low |
| Community-based, aged-care interventions Luker et al. (2019) | 1 | 18 | RCT | Serious^a^ | Not serious | Not serious | Serious^e^ | Strongly suspected^h^ | Very low |
|  | 1 | 24 | RCT | Serious^b^ | Not serious | Not serious | Very serious^e,f^ | Strongly suspected^h^ | Very low |
| Preventive home visit  Mayo-Wilson et al. (2014) | 7 | 7-12 | RCT | Serious^a^ | Not serious | Not serious | Not serious | Undetected | Moderate |
| CGA in community setting  Briggs et al. (2022) | 2 | 13-24 | RCT | Serious^a^ | Not serious | Not serious | Serious^g^ | Undetected | Low |
| Medication review by pharmacist in LTC  Sadowski et al. (2020) | 2 | 12 | RCT | Very serious^a^ | Not serious | Not serious | Serious^g^ | Strongly suspected^h^ | Very low |
| **Hospital readmission** | | | | | | | | | |
| Caregiver integration during discharge planning  Rodakowski et al. (2017) | 13 | 1-6 | RCT | Not serious | Not serious | Not serious | Not serious | Undetected | High |
| Transitional care programs for community-dwelling older adults  Weeks et al. (2018) | 10 | 1-6 | RCT | Not serious | Serious^c^ | Not serious | Serious^e^ | Strongly detected^h^ | Very low |
|  | 2 | 1-6 | Observational | Not serious | Not serious | Not serious | Not serious | Strongly suspected^h^ | Very low |
| Transitional care programs for LTCF residents  Birtwell et al. (2022) | 11 | 1-6 | Mix | Serious^a^ | Serious^d^ | Not serious | Not serious | Undetected | Very low |
| Integrating primary healthcare in aftercare  Ran Li et al. (2022) | 22 | 1-6 | RCT | Serious^a^ | Serious^c^ | Not serious | Not serious | Undetected | Low |
| Continuity of care  Facchinetti et al. (2022) | 21 | 1-6 | RCT | Not serious | Very serious^c,d^ | Not serious | Not serious | Strongly detected^i^ | Very low |
|  | 12 | 7-12 | RCT | Not serious | Serious^d^ | Not serious | Not serious | Undetected | Moderate |
| Community-based, aged care interventions  Luker et al. (2019) | 1 | 6 | RCT | Serious^a^ | Not serious | Not serious | Serious^g^ | Undetected | Low |
| **Length of stay** | | | | | | | | | |
| Early supported discharge  William et al. (2022) | 4 |  | RCT | Not serious | Very serious^c,d^ | Not serious | Not serious | Strongly suspected^h^ | Very low |
| Perioperative geriatric intervention Thillainadesan et al. (2020) | 8 |  | RCT | Serious^b^ | Not serious | Not serious | Not serious | Undetected | Moderate |
| CGA-ward  Fox et al. (2012) | 7 | 3 | RCT | Very serious^a^ | Serious^d^ | Not serious | Serious^e^ | Strongly suspected^h^ | Very low |
| **Emergency department utilisation** | | | | | | | | | |
| **ED visit** | | | | | | | | | |
| Community-based case management  Poupard et al. (2019) | 1 | 12 | RCT | Not serious | Not serious | Not serious | Very serious^e,f^ | Strongly suspected^h^ | Very low |
| CGA in community setting  Briggs et al. (2022) | 1 | 12 | RCT | Serious^a^ | Not serious | Not serious | Serious^g^ | Undetected | Low |
| **ED LOS** | | | | | | | | | |
| Transitional care programs for LTCF residents  Birtwell et al. (2022) | 3 | NR | Observational | Very serious^a^ | Very serious^c,d^ | Not serious | Not serious | Strongly suspected^h^ | Very low |
| **Drug use** | | | | | | | | | |
| Anti-microbial stewardship  Crespo-Rivas et al. (2021) | 3 | 12 | RCT | Not serious | Serious^c^ | Not serious | Serious^f^ | Strongly suspected^i^ | Very low |
| **Primary care visit** | | | | | | | | | |
| Community-based, aged care interventions  Luker et al. (2019) | 1 | NR | RCT | Very serious^a^ | Not serious | Not serious | Not serious | Strongly suspected^h^ | Very low |

a: high RoB; PEDro (6-5 score); Cochrane (5-6 domain as high risk/unclear); stated in paper as high

b: moderate RoB; PEDro (4 n below score); Cochrane (3-4 domain as high risk/unclear); stated in paper as moderate/some concern

c: high heterogeneity across one of studies

d: wide variation in effect estimates

e: wide 95%CI

f: total number of participants <400

g: total number of events <300

h: no reports on publication bias

i: funnel plot asymmetry
